# Supplementary material for: Evaluating the sustainability of differentiated service delivery interventions for stable ART clients in sub-Saharan Africa: a systematic review protocol
Source: BMJ Open. 2020 Feb 2;10(1):e033156. doi: 10.1136/bmjopen-2019-033156 (PMC7045032; doi:10.1136/bmjopen-2019-033156)
Supplement: Supplementary data [file bmjopen-2019-033156supp001.pdf]

Supplementary File I: Search strategy (PubMed & EMBASE)

**#1 Pubmed Search ALL <2000 to November 21, 2019>**	
**Search history sorted by search number ascending**	
**#**	**Searches**
**1**	(patient-centred care or patient-centered care or community supported models or adherence clubs or teen* club or task shifting or ("community ART" adj2 (distribution or delivery or refill)) or community client lead ART-delivery or facility fast track or quick pick-up or (p?ediatric expedited adj2 ART)).mp. or ((differentiated or decentrali?ed or community) adj2 (care or intervention)).ti,ab,kf. or (adherence club or differentiated model* or down-referr* or out-of-clinic).ti,ab,kf.
**2**	exp HIV/ or exp HIV INFECTIONS/ or exp HIV Seropositivity/ or hiv.ti,ab,kf. or (antiretroviral treatment or ART).ti,ab,kf.
**3**	1 and 2
**#2 Embase search <2000 to November 21, 2019>**	
**Search history sorted by search number ascending**	
**1**	(patient-centred care or patient-centered care or community supported models or adherence clubs or teen* club or task shifting or ("community ART" adj2 (distribution or delivery or refill)) or community client lead ART-delivery or facility fast track or quick pick-up or (p?ediatric expedited adj2 ART)).mp. or ((differentiated or decentrali?ed or community) adj2 (care or intervention)).ti,ab,kw. or (adherence club or differentiated model* or down-referr* or out-of-clinic).ti,ab,kw.
**2**	exp Human immunodeficiency virus/
**3**	exp Human immunodeficiency virus infection/
**4**	(hiv or (antiretroviral treatment or ART)).ti,ab,kw.
**5**	2 or 3 or 4
**6**	1 and 5
**7**	limit 6 to (conference abstracts or embase)
