# Supplementary material for: Evaluating the sustainability of differentiated service delivery interventions for stable ART clients in sub-Saharan Africa: a systematic review protocol
Source: BMJ Open. 2020 Feb 2;10(1):e033156. doi: 10.1136/bmjopen-2019-033156 (PMC7045032; doi:10.1136/bmjopen-2019-033156)
Supplement: Supplementary data [file bmjopen-2019-033156supp002.pdf]

Supplementary File II: Data extraction tool

Publication detail		Setting		Intervention detail																
Author, Year, Journal	Title of publication	Town, Country e.g. Tete, Mozambique	Setting i.e. Rural or Urban	HIV project/Study site e.g. MSF project	Project ownership i.e. Government (MOH)or NGO (MSF)	Year of commencement of Intervention	Who is the Target population/ Intervention group	Comparison group/contemporary or historical	Definition of "Stable" patient	Number of participants (stable)	Number of participants (comparison)	Model e.g. Decentralized care, DCM (if so which? Task shifting	Brief description of intervention	Level of implementation (number of sites)	Study aim	Study variables definitions	Primary care provider in intervention	Primary care provider in standard of care (SOC)	Provider in intervention funded by	Provider in SOC funded by

Study detail		Quantitative Outcomes - any numeric patient-centered outcomes to show effectiveness (*Insert reported outcome values/units/definition only*)																							
Study design	Any Theoretical underpinning for the study	Outcomes - VL (%) SOC	Outcomes - VL (%) DCM intervention	Outcome - VL (rate) SOC	Outcome - VL (rate) DCM intervention	*Comments about units or subgroup analysis*	Outcome - Retention (%)SOC	Outcome - Retention (%)DCM intervention	*Comments about units or any subgroup analysis*	Outcome - LTFU (%)SOC	Outcome - LTFU (%)DCM intervention	Outcome - LTFU (rate) SOC	Outcome - LTFU (rate)	*Comments about units or any subgroup analysis*	Outcome - Mortality (%) SOC	Outcome - Mortality (%) DCM intervention)	Outcome - Mortality (rate)SOC	Outcome - Mortality (rate)DCM intervention	*Comments about units or any subgroup analysis*	Outcome - Provider Cost/visit ($)SOC	Outcome - Provider Cost/visit ($)DCM intervention)	Outcome - Patient. Cost/visit ($)SOC	Outcome - Patient. Cost/visit ($)DCM intervention)	*Comments about units or any subgroup analysis*

Intervention Design and Delivery								External environment				Intervention Process					
**Evidence base** - Is there evidence that the intervention provides the expected benefits as planned i.e. that the DSD improves outcomes	**Expertise** - Is there evidence of adequate expert knowledge and experience to carry out the DSD especially by supporting organisation	**QI methods** - Is there evidence that QI methods i.e. using data to identify gaps which are continually improved, starting with a pilot and then spreading etc. are used to support intervention success and sustainability	**Monitoring progress** - Is there a standardized and systematic method to gather and report data during DSD intervention	**Project duration** - Is there evidence that the intervention will last beyond initial funding	**Project type** - What type of intervention is it e.g. prevention, treatment, palliative, supportive care	**Problem** - Is there general awareness of a problem among stakeholders that requires the DSD intervention to address	**Capacity building** - Is there evidence of any orientation, training, on-going mentoring for staff delivering the DSD intervention	**Awareness** - Is there evidence of the larger community being aware of the DSD intervention and promoting its benefit	**Political support** - Is there evidence that the intervention has political support? e.g. government engagement, guidelines revision to include DSD requirement etc.	**Spread** - Is there evidence that the intervention or underlying concepts spread within participating organisation or to other locations	**Urgency** - Is there evidence of an urgency to maintain the DSD intervention based on its relevance	**Roles & responsibilities** - Is there evidence that roles & responsibilities of staff involved in the DSD are spread out and clearly defined	**Belief in initiative** - Is there evidence that staff think the DSD intervention is a better way to do things	**Complexity** - Is there evidence that it is not difficult for staff to understand and conduct the DSD intervention	**Shared goal** - Is there evidence of a shared aim and vision established with all stakeholders before commencing the DSD intervention	**Incentives** - Is there evidence that rewards or benefits derived from the DSD intervention are considered enough motivation that drive stakeholders to engage and continue delivering intervention over time	**Job requirement /descriptions** - Is there evidence of revision of job requirement for key staff incorporating the DSD intervention tasks as part of key job descriptions
3 = There is sufficient evidence that the intervention provides expected benefits to stakeholders	3 = There is sufficient evidence that the supporting organization has adequate expert knowledge and experience to conduct intervention	3 = There is sufficient evidence of the use of QI methods to support the conduct of intervention	3 = There is sufficient evidence of monitoring the intervention using standardised system to gather and report data over time	3 = There is sufficient evidence the intervention will last for a long time beyond the initial project and funding	3 = The type and design of the project is clear (e.g. preventive, treatment, palliative care, supportive care etc.)	3 = The intervention is addressing a problem that is recognised and accepted as a real concern by all stakeholders i.e. community, staff, patients, supporting organisation, facility, government	3 = There is sufficient evidence of orientation/ training/ mentoring to new staff and on-going training to all staff to be able to deliver intervention successfully	3 = There is sufficient evidence of effort to ensure that stakeholders e.g. the community are aware of the benefits of the intervention through media marketing e.g. patient pressure groups, community leaders	3 = There is sufficient evidence the intervention has the full support of Government (involvement of the MOH, District/State/zonal health unit, institutions, Revision of guideline, inclusion in strategic plans etc.)	3 = There is sufficient evidence that the intervention or beneficial parts of it are spread within a facility or to other facilities in a community or district	3 = There is sufficient evidence of motivation or urgency to maintain the intervention or parts of it based on its perceived potential of supporting a relevant healthcare need.	3 = There is sufficient evidence that roles and responsibilities of all staff involved in the intervention is clear and evenly distributed so no staff is over-burdened	3 = There is sufficient evidence that majority of staff conducting the intervention believe the change is a better way of doing thing and will add value	3 = There is sufficient evidence that it is not hard to understand, conduct and maintain the intervention	3 = There is sufficient evidence of a shared aim and vision for the intervention existing among all major stakeholders including the community, government, partners, patients as well as goal revision when necessary	3 = There is sufficient evidence of perceived benefit from the intervention by all stakeholders including community, supporting organisation, patients, staff and government	3 = There is sufficient evidence of revised job requirement for key staff in facilities which capture the roles and job functions introduced by the intervention e.g. revised job description, SOP, guidelines OR a revision in job requirement was not required
2 = There is some evidence that the intervention provides expected benefits to stakeholders	2 = There is some evidence that the supporting organisation has adequate expert knowledge and experience to conduct intervention	2 = There is some evidence of the use of QI methods to support the conduct of intervention	2 = There is some evidence of monitoring the intervention using standardised system to gather and report data over time	2 = There is some evidence the intervention will last for some time beyond the initial project and funding	2 = The type and design of the project is clear to some extent (e.g. preventive, treatment, palliative care, supportive care etc.)	2 = The intervention is addressing a problem that is recognised and accepted as a concern by some stakeholders i.e. mainly by the facility, patients, staff, supporting organization	2 = There is some evidence of orientation/training/ mentoring to new staff and to all staff to be able to deliver intervention successfully	2 = There is some evidence of effort to ensure that stakeholders e.g. the community are aware of the benefits of the intervention through media marketing e.g. community leaders alone or patients or pressure groups	2 = There is evidence the intervention has some level of support of the Government (involvement of the MOH, District/State/zonal health unit, an institution, etc.)	2 = There is some evidence the intervention spread to a few other sites beyond the intervention facility	2 = There is some evidence of motivation or urgency to maintain the intervention or parts of it based on its perceived potential of supporting a relevant healthcare need.	2 = There is some evidence that roles and responsibilities of staff involved in the intervention is clear and evenly distributed so no staff is over-burdened	2 = There is some evidence that staff conducting the intervention believe the change is a better way of doing thing and will add value	2 = There is evidence of some difficulty in understanding or conducting and maintaining the intervention	2 = There is some evidence of a shared aim and vision for the intervention existing among most stakeholders including the community, a local partners and patients.	2 = There is some evidence of perceived benefit from the intervention by some stakeholders e.g. only supporting organisation or patients or staff	2 = There is some evidence of revised job requirement for key staff in facilities with new roles and job functions introduced by the intervention but no revised job descriptions, SOP
1 = There is little or no evidence that the intervention provides expected benefits to stakeholders	1 = There is little or no evidence the supporting organisation has adequate expert knowledge and experience to conduct intervention	1 = There is little or no evidence of the use of QI methods to support the conduct of intervention to a little or no extent	1 = There is little or no evidence of monitoring the intervention using standardised system to gather and report data over time	1 = There is little or evidence the intervention will last beyond the initial project and funding	1 = The type and design of the project is clear to a little extent (e.g. preventive, treatment, palliative care, supportive care etc.)	1 = The intervention is addressing a problem that is not relly recognised and accepted as a concern by any stakeholders	1 = There is little or no evidence of orientation/training/ mentoring to staff to be able to deliver intervention successfully	1 = Only PLHIV and lay workers in the community participating in the intervention are aware of the intervention	1 = There is evidence the intervention has the support of at least the institution involved in implementation, a local NGO etc.	1 = There is evidence the intervention or beneficial parts of it are spread to at least other parts of a facility	1 = There is little or no evidence of motivation or urgency to maintain the intervention or parts of it based on its perceived potential of supporting a relevant healthcare need.	1 = There is little or no evidence that roles and responsibilities of staff involved in the intervention are clear or evenly distributed	1 = There is little or no evidence that staff conducting the intervention believe the change is a better way of doing things	1 = There is evidence of moderate difficulty in understanding or conducting and maintaining the intervention	1 = There is little or no evidence of a shared aim and vision for the intervention existing among stakeholders. Only the supporting partner developed a goal.	1 = There is little or no evidence of perceived benefit from the intervention by any stakeholder, maybe only the supporting organisation	1 = There is little or no evidence of revised job requirement for the staff invloved with implementing the intervention at the facilities

	Resources				
**Additional workload** - Is there evidence that any additional workload introduced by the DSD intervention is manageable and requiring no special effort to staff involved	**Resources - General** - Is there evidence that resources needed to manage and maintain the DSD intervention are available	**Funding** - Is there evidence that adequate funds are available to implement and strategic funds planned to sustain the DSD intervention	**Infrastructure** - Is there evidence that resources required to support the DSD intervention e.g. office space, materials, and supplies are available	**Staff** - Is there evidence of sufficient staff in place to conduct and sustain the DSD intervention	**Time** - Is there evidence that adequate time was dedicated for the DSD intervention in the routine daily schedule of the facility
3 = There is sufficient evidence that any additional workload introduced by the intervention is manageable and evenly divided among staff without requiring extra effort	3 = There is sufficient evidence that all/most resources required to conduct and maintain the intervention are available and adequate and provided by the government i.e. more government less external donor	3 = There is sufficient evidence that adequate (all/most) funds required to implement and sustain the intervention are available and provided by government	3 = There is sufficient evidence that all/most of the resources required to support the intervention such as buildings, office space, materials and supplies are available and provided by the government	3 = There is sufficient evidence of sufficient number of staff, internal and external (i.e. a team) in place to implement and sustain the intervention employed by the government	3 = There is sufficient evidence that adequate time was dedicated to the intervention activities in the routine daily schedule of the facility
2 = There is evidence that the additional workload introduced by the intervention is manageable to a some extent and evenly divided among staff	2 = There is evidence that some resources required to conduct and maintain the intervention are available and adequate i.e. less government and more external donor	2 = There is evidence that most of the funds required to implement and sustain the intervention are available e.g.less government funds and more external donor funds	2 = There is evidence that the resources required to support the intervention such as buildings, office space, materials and supplies are available i.e. less government support and more external donor support	2 = There is evidence that the number of internal staff in place to implement and sustain the intervention is mostly adequate i.e. the cadre of staff needed is employed by govenrment but paid with external donor funds	2 = There is evidence that time was dedicated to some of the intervention activities in the routine daily schedule of the facility
1 = There is little or no evidence that the additional workload introduced by the intervention is manageable to the staff involved	1 = There is evidence that little or none of the resources required to conduct and maintain the intervention are available and adequate i.e. mostly provided by external donor	1 = There is evidence that little or none of the funds required to implement and sustain the intervention are available e.g.mostly provided by external donor funds	1 = There is evidence that the resources required to support the intervention such as buildings, office space, materials and supplies are available and mostly paid by external funder	1 = There is evidence that the number of internal staff in place to implement and sustain the intervention is somewhat adequate i.e. project staff are mainly employed and paid by extarnal funder	1 = There is evidence that time was dedicated to little or none of the intervention activities in the routine daily schedule of the facility i.e. project activities run within the facility but parallel to routine facility activities

Organizational setting					
**Integration** - Is there evidence that the DSD intervention was embedded within the existing organizational structure, Programme and policies	**Adaptation** - Is there evidence that the DSD intervention is flexible to respond, change, adapt and fit with local context requirement	**Opposition** - Is there evidence of any resistance due to other competing interests from stakeholders reported	**Readiness** - Is there evidence that health facilities have adequate capacity and readiness to undertake the DSD intervention i.e. in terms of materials and manpower	**Organizational Value system and culture** - Is there evidence that the values of the DSD intervention align with health system values, prevailing beliefs and culture and priorities	**Management support** - Is there evidence of facility management support for the delivery and maintenance of the DSD intervention
3 = There is sufficient evidence that the intervention was embedded within the existing organizational structures, programmes and policies of the health system and the facility	3 = There is sufficient evidence that the intervention responds to changes and adapts to fit with local context and requirements	3 = There is sufficient evidence of no resistance from stakeholders to the intervention due to other competing priorities	3 = There is sufficient evidence of adequate capacity and readiness of facilities to conduct the intervention i.e. all materials and staff needed are provided by government	3 = There is sufficient evidence that the health system and facility values, prevailing beliefs and culture and priorities support the sustainability and strategic direction of the intervention e.g. inclusion instragegic plan or guideline	3 = There is sufficient evidence of management total support of the intervention in the form of reminders, staff, technical and education to enhance delivery
2 = There is evidence that the intervention was embedded to some extent within the existing organizational structures, programmes and policies of the facility	2 = There is some evidence that the intervention responds to changes and adapts to fit with local context and requirements	2 = There is evidence of some resistance from stakeholders to the intervention due to other competing priorities	2 = There is some evidence of capacity and readiness of facilities to conduct the intervention i.e. most materials and staff needed are provided by government with support from external funder	2 = There is some evidence that the health system and facility values, prevailing beliefs and culture and priorities support the sustainability and strategic direction of the intervention e.g. some government involvement with little commitment	2 = There is some evidence of management support of the intervention to a large extent in the form of reminders, staff, technical and education to enhance delivery i.e. in principle but it is non-commital
1 = There is evidence that the intervention was embedded to a little extent within the existing organizational structures, programmes and policies of the facility	1 = There is little or no evidence that the intervention responds to changes and adapts to fit with local context and requirements	1 = There is evidence of resistance to a large extent from stakeholders to the intervention	1 = There is evidence that capacity and readiness of facilities to conduct the intervention is limited i.e. aii/most materials and staff needed are provided by external funder	1 = There is little or no evidence that the health system and facility values, prevailing beliefs and culture and priorities support the sustainability and strategic direction of the intervention	1 = There is little or no evidence of management support of the intervention

People Involved								
**Leaders/champions** - Is there evidence of any influential person or group who advocates and supports the DSD intervention	**Ownership** - Is there evidence that stakeholders take ownership to support, embed and sustain the DSD intervention	**Power** - Is there evidence that stakeholders have the ability to use their power to make decisions, advocate and support the DSD intervention	**Collaboration** - Is there evidence of any collaborations, partnerships and support networks to promote and sustain the DSD intervention	**Satisfaction** - Is there evidence of benefits and rewards enjoyed by stakeholders and staff for participation in the DSD intervention reported	**Stakeholders participation** - Is there evidence that key stakeholders (those affected by the intervention) are engaged and participate in the DSD intervention	**Community participation** - Is there evidence of the participation of community members in directing and shaping DSD intervention goals and approaches to reflect their values and needs	**Patient involvement** - Is there evidence of the involvement of patients in DSD intervention processes to understand patient's perspectives, values and needs	**Staff involvement** - Is there evidence of the involvement of staff in the planning, design, delivery of the DSD intervention
3 = There is evidence of an influential person (champion) and group of people (patient pressure group) who have the ability and skills to advocate, communicate and support the intervention e.g. a prominent community leader, a PLHIV group, NGO	3 = There is sufficient evidence that the government, facilities, communities and other stakeholders take ownership and responsibility to support the intervention	3 = There is sufficient evidence of the ability of stakeholders to use their power to make decisions, advocate and support initiative	3 = There is sufficient evidence of partnerships, collaborations and networks to support and sustain the intervention e.g. with government and other local stakeholders, patient groups etc.	3 = There is sufficient evidence of acceptance, enjoyment and reward among stakeholders from participating in intervention e.g. Government, local partners, staff, patients etc.	3 = There is sufficient evidence of the involvement and participation of stakeholders who are affected by the intervention e.g. Government, community, staff, patients	3 = There is sufficient evidence of the participation of community members to direct and shape the intervention to reflect their values, expectations and needs e.g. involving community groups and leaders	3 = There is sufficient evidence of the involvement of patients in the intervention's processes, to understand patient's perspective i.e. in the design and process	3 = There is sufficient evidence of the involvement of staff in the planning, design, delivery and maintenance of the intervention
2 = There is some evidence of an influential person (champion) and group of people (patient pressure group) who have the ability and skills to advocate, communicate and support the intervention e.g. expert patients living openly with their status	2 = There is some evidence that the facilities and other stakeholders i.e. the community or any local partner take ownership and responsibility to support the intervention	2 = There is some evidence that stakeholders have the ability to use their power to make decisions, advocate and support initiative	2 = There is evidence of some level of partnerships, collaborations and networks to support and sustain the intervention e.g. with some local stakeholders	2 = There is evidence of some level of acceptance, enjoyment and reward among stakeholders from participating in intervention e.g. Among direct beneficiaries staff and patients	2 = There is evidence of some level of involvement and participation of stakeholders who are affected by the intervention e.g. staff, patients	2 = There is evidence of some level of participation of community members to direct and shape the intervention to reflect their values, expectations and needs involving community groups e.g. involving lay workers from the community	2 = There is evidence of some level of involvement of patients in the intervention's processes, to understand patient's perspective e.g. adapting the process	2 = There is evidence of some level of involvement of staff in the planning, design, delivery and maintenance of the intervention
1 = There is little or no evidence of an influential patient, or group of people who have the ability and skills to advocate, communicate and support other patients at the facility level during the intervention.	1 = There is little or no evidence that any stakeholder take ownership and responsibility to support the intervention maybe just the facilities	1 = There is little or no evidence of the ability of stakeholders to use their power to make decisions, advocate and support initiative	1 = There is little or no evidence of partnerships, collaborations and networks to support and sustain the intervention e.g. with the facility or institution (s)	1 = There is little or no evidence of acceptance, enjoyment and reward among any stakeholder from participating in intervention	1 = There is little or no evidence of the involvement and participation of stakeholders who are affected by the intervention e.g. patients	1 = There is little or no evidence of participation of community memberst o direct and shape the intervention to reflect their values, expectations and needs involving community groups e.g. community only involved as passive recipients	1 = There is little or no evidence of the involvement of patients in the intervention's processes e.g. only as passive participants	1 = There is evidence of involvement of staff in the delivery and maintenance of the intervention

Challenges reported	Comments (Pros & Cons)	Patients perspective	Staff perspective
Any challenges documented during implementation	Any gains or flaws highlighted by staff or patients during implementation	Any experiences or opinions expressed by patients	Any experiences or opinions expressed by staff
