# Supplementary material for: Evaluating the sustainability of differentiated service delivery interventions for stable ART clients in sub-Saharan Africa: a systematic review protocol
Source: BMJ Open. 2020 Feb 2;10(1):e033156. doi: 10.1136/bmjopen-2019-033156 (PMC7045032; doi:10.1136/bmjopen-2019-033156)
Supplement: Author's manuscript [file bmjopen-2019-033156.draft_revisions.pdf]

BMJ Open

BMJ Open is committed to open peer review. As part of this commitment we make the peer review history of every article we publish publicly available.

When an article is published we post the peer reviewers' comments and the authors' responses online. We also post the versions of the paper that were used during peer review. These are the versions that the peer review comments apply to.

The versions of the paper that follow are the versions that were submitted during the peer review process. They are not the versions of record or the final published versions. They should not be cited or distributed as the published version of this manuscript.

BMJ Open is an open access journal and the full, final, typeset and author-corrected version of record of the manuscript is available on our site with no access controls, subscription charges or pay-per-view fees (http://bmjopen.bmj.com).

If you have any questions on BMJ Open's open peer review process please email info.bmjopen@bmj.com

BMJ Open

Evaluating the sustainability of differentiated service delivery interventions for stable ART patients in sub-Saharan Africa: A systematic review protocol

Journal:	*BMJ Open*
Manuscript ID	bmjopen-2019-033156
Article Type:	Protocol
Date Submitted by the Author:	23-Jul-2019
Complete List of Authors:	Okere, Nwanneka; Amsterdam Institute for Global Health and Development, Global Health, University of Amsterdam<br>Urlings, Lisa; Amsterdam University Medical Centres<br>Naniche, Denise; Barcelona Institute for Global Health, University of Barcelona<br>Rinke de Wit, Tobias; Amsterdam Institute for Global Health and Development, Global Health, University of Amsterdam<br>Gomez, Gabriela; London School of Hygiene and Tropical Medicine,<br>Hermans, Sabine; Amsterdam Institute for Global Health and Development, Global Health, University of Amsterdam
Keywords:	Sustainability, Differentiated, Stable, Constructs

SCHOLARONE™
Manuscripts

Evaluating the sustainability of differentiated service delivery interventions for stable ART patients in sub-Saharan Africa: A systematic review protocol

Nwanneka E Okere[1] (0000-0001-9182-6518), Lisa Urlings[2], Denise Naniche[3], Tobias F Rinke de Wit[1], *Gabriela B Gomez[4], *Sabine Hermans[1]

[1] Amsterdam Institute for Global Health and Development, Department of Global Health, Amsterdam UMC, University of Amsterdam, Netherlands
[2] Amsterdam UMC University of Amsterdam Netherlands
[3] Barcelona Institute for Global Health, University of Barcelona, Barcelona, Spain
[4] Department of Global Health and Development London School of Health and Tropical Medicine UK

*These authors share senior authorship

[**Correspondence**: Nwanneka Ebelechukwu Okere (0000-0001-9182-6518), Amsterdam Institute for Global Health and Development AHTC Tower C4 Paasheuvelweg 25 1105 BP Amsterdam, The Netherlands, n.okere@aighd.org, +31 0645991604]

Word count: 2128 excluding title page, abstract, references and tables

Keywords: Sustainability, Differentiated, Constructs, Stable, Funding, Processes

For peer review only - http://bmjopen.bmj.com/site/about/guidelines.xhtml

ABSTRACT

Introduction: In 2015, the WHO recommended immediate treatment for people living with HIV (PLHIV), so called 'universal test and treat'. As a result, the number of PLHIV needing antiretroviral therapy (ART) in sub-Saharan Africa (SSA) doubled from 12 million to >25 million. This strain on already weak health systems inspired the exploration of innovative service delivery models beyond the traditional clinic-based care. Differentiated service delivery (DSD) tailors services according to patient type and offers the much-needed improvement in efficiency. The potential of achieving good outcomes for both patients and the health system plus the promise of sustainability motivates DSD promotion especially in low- and medium-income countries (LMIC). This review aims to evaluate the sustainability of DSD interventions.

Methods and analysis: We will systematically review the literature by selecting peer-reviewed English literature published between 2000 and 2019. Our searches and inclusion/exclusion criteria will be applied to find and select studies about DSD interventions in SSA that included stable ART patients and compared the results to clinic-based service delivery. We will apply a comprehensive sustainability framework including 40 individual constructs to describe, evaluate, score and rank each intervention for sustainability. Narrative and quantitative synthesis and will be conducted as appropriate.

Ethics and dissemination: No ethical approval is required for this study as it is a review of published or publicly available data. Review results will be published in a peer-reviewed journal and presented at international conferences.

PROSPERO registration number: CRD42019120891

For peer review only

STRENGTHS AND LIMITATIONS

1. The current systematic review will assess retrospectively the sustainability of differentiated service models for HIV treatment to date, providing essential information for evaluation and implementation.
2. The research question we tackle addresses a topical issue for HIV programs especially in SSA. The rapid adoption and expansion of DSD interventions by countries warrants an evaluation of their sustainability. This review will equip policy makers with information necessary for making rational choices to prevent wastage of limited resources.
3. A wide range of interventions have been reported and consequently a wide range of outcomes are expected to be included. This could challenge the comparability of sustainability assessments.
4. We propose to review only articles in English, this may prove to bias our review by excluding locally relevant articles in other languages.

For peer review only

For peer review only - http://bmjopen.bmj.com/site/about/guidelines.xhtml

INTRODUCTION

In 2015, the WHO recommended immediate treatment for people living with HIV (PLHIV), so called 'universal test and treat' strategy. As a result, the number of PLHIV needing antiretroviral treatment (ART) in sub-Saharan Africa (SSA) more than doubled from 12 million to over 25 million [1,2]. This increase in demand adds strain on already weak health systems. It made obvious the insufficiency of traditional clinic-based care and calls for innovative service delivery models. Differentiated service delivery (DSD) prioritises patient preferences while also hoping to increase health system efficiency [3–8]. DSD has evolved over time and encompasses a wide range of concepts in HIV programs implementation especially in low- and medium-income countries (LMIC) such as task shifting, decentralization, community-based ART etc. Ultimately, the goal of DSD is to increase access to and retention in care by reducing demand- and supply-side barriers of clinic-based models of service delivery, especially for rural populations [9–11].

Differentiated care became necessary as more patients were initiated on lifesaving antiretrovirals and ART clinics were becoming crowded. This largely influenced the public health approach involving decentralization recommended by WHO as far back as 2006 [12,13]. Since then, attempts have been made to streamline services by categorizing patients in terms of their clinical profile, such as being stable. Currently, WHO defines a stable patient as: "being on ART for at least one year, no current illnesses or pregnancy, a good understanding of lifelong adherence and evidence of treatment success (two consecutive viral load measurements below 1,000 copies/mL)"[1].

Four DSD models were recently described which focus on stable ART patients [14,15]. They include (a) healthcare worker (HCW) groups, (b) facility-based individual models, (c) client managed groups and (d) out-of-facility individual models. Each model is defined by four dimensions, namely 'who' provides care, 'what' is provided, 'when' do the activities happen, and 'where' is care provided. Pre-packaged ART refill, symptoms screening, health talks, clinical consultations or referral if unwell defines 'what' care is provided across models. 'When' care is provided is a major benefit of DSD and is usually less frequent than the routine monthly visits for patients categorized as stable. Though WHO recommends 3-6 months intervals, anything between 2-12 months is possible depending on the context.

More distinctively, these models are characterized by the other two dimensions i.e. 'who' provides care (the cadre of healthcare personnel involved) and 'where' care is provided (location). Interventions may be conducted by HCWs within facilities e.g. adherence clubs (ACs), fast track ART refill (FTA), six-monthly appointments (SMA), multi-month scripting (MMS) or by community based HCWs e.g. community drug distribution points (CDDP). Interventions can also be conducted by non-HCWs within facilities e.g. ACs or by community based non-HCWs e.g. Community ART refill groups (CARG), Community ART groups (CAG), Mobile outreach (OR) and AC. (Table 1)

For peer review only - http://bmjopen.bmj.com/site/about/guidelines.xhtml

Table 1: Characterization of DCM models[16]

		Location (Where)	
		Within facilities	Community based
Personnel (Who)	HCW-managed	Fast track ART refill (FTR)<br>Six monthly appointment (SMA)<br>Multi-month scripting (MMS)<br>Adherence clubs (AC)<br>(Model 1)	Adherence clubs (AC)<br>Community drug distribution points (CDDP)<br><br>(Model 2)
	Non-HCW managed	Adherence clubs (AC)<br><br>(Model 3)	Community ART groups (CAG)<br>Community ART refill groups (CARG)<br>Adherence clubs (AC)<br>Mobile outreach (OR)<br>(Model 4)

The potential of achieving lasting impact and sustainability is the push behind DSD promotion. Sustainability in health care is a broad concept which was only recently comprehensively defined as "program, clinical intervention, and/or implementation strategies which continue to be delivered after a defined period of time (i.e. after expiration of initial external funding) including the maintenance of program or individual behaviour change (i.e., clinician, patient) which may evolve or adapt while continuing to produce benefits for individuals/systems" [17]. This definition consolidates key constructs commonly used to describe sustainability of health interventions in literature.

Several conceptual frameworks have been described articulating constructs associated with sustainability of health interventions [18–24]. Most of them view sustainability as a process to be prospectively explored during implementation rather than an outcome to be retrospectively evaluated. Lennox et al [25], in a recent review, developed a comprehensive framework comprising 40 individual sustainability constructs categorized into six broad domains, namely (1) intervention design and delivery, (2) intervention processes, (3) external environment, (4) organizational setting, (5) resources and (6) people involved.

Why is it important to do this review?

Until a vaccine is developed, the long-term sustainability of HIV programs is not only desirable but essential, as lifelong ART remains the only option for PLHIV. Therefore, sustainability underlies every strategy so far endorsed for HIV programs e.g. task shifting, decentralization of care , differentiation of care [14,26–28]. The increasing call for universal health coverage by the Global Fund to fight AIDS, tuberculosis and malaria and the World Health Organization is similarly motivated and will likely benefit not just HIV programs but entire health systems [29,30]. While many DSD interventions have been implemented within HIV programs with good outcomes, knowledge about their sustainability has been

For peer review only - http://bmjopen.bmj.com/site/about/guidelines.xhtml

limited [31–33]. It is not clear how these interventions can be properly integrated within the existing health system to ensure they last.

Research on the sustainability of health interventions has been hampered by a lack of consensus. For example, several definitions have existed, some referring to the continuation of an intervention as a whole or in part [34] and others to the continuation of benefits of an intervention [35]. Similarly, several sustainability frameworks exist, making comparability across studies difficult. Common grounds are however beginning to emerge. The framework by Shediac-Rizkallah [36], arguably laid the foundation for identifying commonalities by providing the basic constructs on which other sustainability studies continue to build [37–39]. The recent syntheses of a comprehensive definition and a comprehensive framework, both anchoring on this framework, provided useful tools which we have adopted to gain insight into the sustainability of DSD models in this review [17,25].

In resource constrained settings, the allure of DSD interventions lies not just on the promising patient-related outcomes but also the reduced demand on resources it places on health systems without undermining effectiveness. These are thought to make DSD feasible to implement even in LMIC settings. HIV programs will therefore require knowledge synthesized from evidence to inform which DSD options to explore. Our review aims to fill this gap by evaluating the sustainability status of available DSD interventions.

METHODS AND ANALYSIS

Protocol registration

This review protocol has been registered in the PROSPERO database (CRD42019120891). The protocol adheres to the Preferred Reporting Items for Systematic Reviews and Meta-Analyses-Protocols (PRISMA-P) 2015 guidelines for preparing protocols of systematic reviews [40].

Search strategy

We will search PubMed and EMBASE to identify eligible articles using a comprehensive search strategy (see Supplementary File I). Additionally, the reference lists of included articles and the reference lists of previously published reviews will be mined for other relevant articles. The review period of interest will be between 2000 and 2019 to capture all articles published since the widespread scale-up of ART programs across SSA in the 2000s. Only English language articles will be included. We will use Rayyan QCRI, the free systematic reviews web application [41] to store, organise and manage eligible references whose titles and abstracts will be screened. A follow-up search will be conducted prior to the completion of the review which is planned nine months after commencement.

Eligibility criteria

We will include observational, experimental or quasi-experimental studies. Reviews, editorials, protocol studies and clinical guidelines will be excluded. We will include studies involving stable adult ART patients accessing HIV care in SSA. The definition of a stable patient has progressed over time, therefore, there will be no restriction in terms of definition. However, patients must have accessed HIV services through other delivery models than standard clinic-based care. The performance of the other

For peer review only - http://bmjopen.bmj.com/site/about/guidelines.xhtml

service delivery model can be compared with standard clinic-based HIV service delivery accessed by other patients. Lack of this comparison is not an exclusion criterion.

Selection of studies

Two reviewers (NEO and UL) will independently screen the titles and abstracts of articles identified in the searches and come up with a list of potential articles. The reviewers will then conduct full-text reading of these articles to determine eligibility. GBG and SMH will be engaged in discussions to make a final determination on eligibility whenever the two reviewers are unable to reach a consensus. Reasons for exclusions will be reported.

Data extraction

We have developed a data extraction form. NEO and UL will pilot the data extraction form on a subset of five articles to assess its functionality and suitability. After incorporating feedback obtained from the review team (NEO, UL, GBG, SMH) a final form will then be used to extract relevant data independently by the two reviewers.

Data to be extracted will include (a) details of each intervention including site, setting, service delivery model, funding, providers, period, theoretical underpinning; (b) study population, participant type and numbers; (c) sustainability constructs as described in Table 2; (d) challenges or un-intended consequences faced implementing the respective DSD, Comments, patients and Staff perspectives (see Supplementary File II).

For peer review only

For peer review only - http://bmjopen.bmj.com/site/about/guidelines.xhtml

Table 2: Sustainability constructs and evidence to be extracted from included papers (adapted from [25]) (Primary study outcomes)

**Domain**	Sustainability construct	Outcome	Evaluation Question
The Intervention Design and Delivery	Demonstrating effectiveness	Patient-related outcomes	Does the paper report any numeric or subjective patient-centred outcomes to show effectiveness e.g. retention-in-care, viral suppression, loss-to-follow-up, patient satisfaction
	Evidence base for the intervention	Evidence base	Is there evidence that the intervention provides the expected benefits as planned i.e. that the DSD improves outcomes
	Expertise	Expertise	Is there evidence of adequate expert knowledge and experience to carry out the DSD especially by supporting organisation
	Quality Improvement (QI)methods	QI methods	Is there evidence that QI methods i.e. using data to identify gaps which are continually improved, starting with a pilot and then spreading etc. are used to support intervention success and sustainability
	Monitoring progress	Monitoring progress	Is there a standardized and systematic method to gather and report data during DSD intervention
	Intervention duration	Duration	Is there evidence that the intervention will last beyond initial funding
	Intervention type	Project Design	What type of intervention is it e.g. prevention, treatment, palliative, supportive care
	The problem	Problem awareness	Is there general awareness of a problem among stakeholders that requires the DSD intervention to address
	Training & Capacity building	Capacity building	Is there evidence of any orientation, training, on-going mentoring for staff delivering the DSD intervention
The External Environment	Awareness and Raising the profile	Community awareness	Is there evidence of the larger community being aware of the DSD intervention and promoting its benefit
	Socioeconomic and political considerations	Political support	Is there evidence that the intervention has political support? e.g. government engagement, guidelines revision to include DSD requirement etc.
	Spread to other organizations	Spread	Is there evidence that the intervention or underlying concepts spread within participating organisation or to other locations
	Urgency	Urgency	Is there evidence of an urgency to maintain the intervention based on its relevance
Intervention processes	Accountability of roles and responsibilities	Roles & responsibilities	Is there evidence that roles & responsibilities of staff involved in the DSD are spread out and clearly defined

For peer review only - http://bmjopen.bmj.com/site/about/guidelines.xhtml

	Belief in the intervention	Belief in intervention	Is there evidence that staff think the DSD intervention is a better way to do things
	Complexity	Complexity	Is there evidence that it is not difficult for staff to understand and conduct the intervention
	Defining Aims and Shared Vision	Shared goal	Is there evidence of a shared aim and vision established with all stakeholders before commencing the intervention
	Incentives	Motivation	Is there evidence that rewards, or benefits derived from the DSD intervention are considered enough motivation that drive stakeholders to engage and continue delivering intervention over time
	Job requirements	Job requirements	Is there evidence of revision of job requirement for key staff incorporating the DSD intervention tasks as part of key job descriptions
	Workload	Workload	Is there evidence that any additional workload introduced by the DSD intervention is manageable and requiring no special effort to staff involved
Resources	General resources	General resources	Is there evidence that resources needed to manage and maintain the DSD intervention are available
	Funding	Funding	Is there evidence that adequate funds are available to implement, and strategic funds planned to sustain intervention i.e. DSD will be embedded and sustained
	Infrastructure	Infrastructure	Is there evidence that resources required to support the DSD intervention e.g. office space, materials, and supplies are available
	Staff	Staff	Is there evidence of enough staff in place to conduct and sustain DSD intervention
	Time	Time	Is there evidence that adequate time was dedicated for DSD intervention in the routine daily schedule of the facility
Organizational setting	Integration with existing programs and policies	Integration	Is there evidence that DSD intervention was embedded within the existing organizational structure, Programme and policies
	Intervention adaptation and receptivity	Adaptation	Is there evidence that the DSD intervention is flexible to respond, change, adapt and fit with local context requirement
	Opposition	Opposition	Is there evidence of any resistance due to other competing interests from stakeholders reported

For peer review only - http://bmjopen.bmj.com/site/about/guidelines.xhtml

	Organizational readiness and capacity	Readiness	Is there evidence that health facilities have adequate capacity and readiness to undertake the DSD intervention i.e. in terms of materials and manpower
	Organizational values and culture	Values & culture	Is there evidence that the values of the DSD intervention align with health system values, prevailing beliefs and culture and priorities
	Support available	Management support	Is there evidence of facility management support for the delivery and maintenance of the DSD intervention
The people involved	Leadership and Champions	Champions	Is there evidence of any influential person or group who advocates and supports the DSD intervention
	Ownership	Ownership	Is there evidence that stakeholders take ownership to support, embed and sustain the DSD intervention
	Power	Power	Is there evidence that stakeholders can use their power to make decisions, advocate and support the DSD intervention
	Relationships, collaboration, networks	Collaboration	Is there evidence of any collaborations, partnerships and support networks to promote and sustain the DSD intervention
	Satisfaction	Satisfaction	Is there evidence of benefits and rewards enjoyed by stakeholders and staff for participation in DSD intervention reported
	Stakeholder participation	Stakeholder participation	Is there evidence that key stakeholders (those affected by the intervention) are engaged and participate in DSD intervention
	Community participation	Community participation	Is there evidence of the participation of community members in directing and shaping DSD intervention goals and approaches to reflect their values and needs
	Patient involvement	Patient involvement	Is there evidence of the involvement of patients in DSD intervention processes to understand patient's perspectives, values and needs
	Staff involvement	Staff involvement	Is there evidence of the involvement of staff in the planning, design, delivery of the DSD intervention

For peer review only - http://bmjopen.bmj.com/site/about/guidelines.xhtml

Data Analysis

Primary outcome measures will include the scores and rankings on sustainability constructs and domains as defined by the Lennox et al framework [25] and presented in Table 2. Since the review aims to extract and score the certainty of evidence retrospectively for sustainability constructs, we simplified a method from a previously described tool to assess sustainability in on-going projects [42]. The two reviewers will assign a score of 3, 2 or 1 independently based on their assessment of whether there was sufficient, some or no evidence about each construct (see Supplementary file II). Additionally, "not described" (ND) will be assigned if the reviewers are not able to make an assessment with information provided. Attempts will be made to contact study authors directly for any additional information or data considered necessary. Secondary outcome measures will include any (a) trends observed in sustainability scores as a result of variations in the criteria employed to define stable patients, (b) if appropriate, any quantitative intervention outcome measures such as retention in care, viral suppression, loss-to-follow-up, patient or provider costs etc. reported across a reasonable number of similar interventions, and (c) qualitative outcomes such as perspectives of patients on issues e.g. satisfaction with intervention or HCW, access to ART, peer support) and perspectives of HCWs on issues e.g. workload, care for sick patients. Qualitative outcomes will be extracted as text which will be summarised using narrative synthesis to identify commonly occurring themes across interventions.

Qualitative and quantitative analyses will be conducted. For qualitative analysis, we will conduct narrative synthesis [43] on all included studies. The basic characteristics, key findings including strengths and challenges of DSD interventions will be summarised. For quantitative analysis, we will employ descriptive statistics to determine the sustainability performance of DSD interventions. Performance will be measured in terms of overall construct scores and percentages and median domain scores. This will then be used to rank constructs, domains, interventions and models for sustainability with higher scores ranked as better sustainability.

Quality of evidence

Most published DSD studies are not randomized controlled trials, therefore, NEO and UL will assess risk of bias in included studies using the Downs and Black checklist [44]. The following domains will be assessed: quality of reporting, external validity and internal validity, confounding (selection bias) and power. For qualitative studies, we will assess risk of bias using the Joanna Briggs Institute (JBI) checklist for qualitative research [45]. Disagreements between reviewers will be resolved as for study selection. The overall quality of included studies will be evaluated, if possible, using the Grading of Recommendations Assessment, Development and Evaluation (GRADE) framework for quality of evidence [46].

ETHICS AND DISSEMINATION

No ethical approval is required for the conduct of this systematic review since all data used are freely available in the public space. Results and findings of the review will be shared published in a peer-reviewed journal and presented at appropriate conferences and meetings.

CONCLUSION

For peer review only - http://bmjopen.bmj.com/site/about/guidelines.xhtml

While DSD interventions may not be as onerous as standard clinical care, they pose some demands to the health system. As countries in SSA strive to increase their share of funding their HIV programs, they must be aware of the aspects influencing sustainability of the DSD options they may select. This review will provide information necessary to inform these discussions. Our review also aims to provide the impetus for further enquiry into service delivery models and socio-economic issues necessary to ensure continual patient empowerment while supporting minimally resourced health systems.

AUTHORS' CONTRIBUTIONS

NEO, GBG and SH conceived the review design and in refining the review. NEO and UL conducted study selection and data extraction. NEO drafted the initial manuscript. All authors contributed to revisions to obtain the final manuscript. All authors read and approved the final manuscript.

PATIENT AND PUBLIC INVOLVEMENT STATEMENT

We will be reviewing and assessing already conducted interventions retrospectively and this will permit little or no involvement of patients and the public.

FUNDING STATEMENT

This work was supported by Erasmus Mundus Joint Doctorate Fellowship, Framework Partnership Agreement 2013-0039, Specific Grant Agreement 2014-0681.

COMPETING INTERESTS' STATEMENT

No competing interests declared

For peer review only

REFERENCES

1 World Health Organization. Consolidated guidelines on the use of antiretroviral drugs for treating and preventing HIV infection: recommendations for a public health approach. *World Heal Organ* 2016;:155 p. doi:10.1016/j.jped.2014.04.007

2 Pustil R. Global AIDS Update. *Aids* 2016;**17 Suppl 4**:S3-11. doi:10.1073/pnas.86.15.5781

3 Bango F, Ashmore J, Wilkinson L, *et al.* Adherence clubs for long-term provision of antiretroviral therapy: cost-effectiveness and access analysis from Khayelitsha, South Africa. *Trop Med Int Heal* 2016;**21**:1115–23. doi:10.1111/tmi.12736

4 Long L, Brennan A, Fox MP, *et al.* Treatment Outcomes and Cost-Effectiveness of Shifting Management of Stable ART Patients to Nurses in South Africa: An Observational Cohort. *PLoS Med* 2011;**8**:e1001055. doi:10.1371/journal.pmed.1001055

5 Babigumira JB, Castelnuovo B, Stergachis A, *et al.* Cost effectiveness of a Pharmacy-only Refill Program in a large Urban HIV/AIDS clinic in Uganda. *PLoS One* 2011;**6**:e18193. doi:10.1371/journal.pone.0018193

6 Grimsrud A, Bygrave H, Doherty M, *et al.* Reimagining HIV service delivery: The role of differentiated care from prevention to suppression: The. J. Int. AIDS Soc. 2016;**19**. doi:10.7448/IAS.19.1.21484

7 Okoboi S, Ding E, Persuad S, *et al.* Community-based ART distribution system can effectively facilitate long-term program retention and low-rates of death and virologic failure in rural Uganda. *AIDS Res Ther* 2015;**12**. doi:10.1186/s12981-015-0077-4

8 Tsondai PR, Wilkinson LS, Grimsrud A, *et al.* High rates of retention and viral suppression in the scale-up of antiretroviral therapy adherence clubs in Cape Town, South Africa. *J Int AIDS Soc* 2017;**20**. doi:10.7448/IAS.20.5.21649

9 Bedelu M, Ford N, Hilderbrand K, *et al.* Implementing Antiretroviral Therapy in Rural Communities: The Lusikisiki Model of Decentralized HIV/AIDS Care. *J Infect Dis* 2007;**196**:S464–8. doi:10.1086/521114

10 Fredlund VG, Nash J. How Far Should They Walk? Increasing Antiretroviral Therapy Access in a Rural Community in Northern KwaZulu-Natal, South Africa. *J Infect Dis* 2007;**196**:S469–73. doi:10.1086/521115

11 Mutevedzi PC, Lessells RJ, Heller T, *et al.* Scale-up of a decentralized HIV treatment programme in rural KwaZulu-Natal, South Africa: does rapid expansion affect patient outcomes? *Bull World Health Organ* 2010;**88**:593–600. doi:10.2471/BLT.09.069419

12 WHO Guidelines. *ANTIRETROVIRAL THERAPY FOR HIV INFECTION IN ADULTS AND ADOLESCENTS: Recommendations for a public health approach HIV/AIDS Programme IMPORTANT: ADDENDUM TO 2006 WHO GUIDELINES ON ANTIRETROVIRAL THERAPY FOR HIV INFECTION IN ADULTS AND ADOLESCENTS*. WHO 2006.

13 Gilks CF, Crowley S, Ekpini R, *et al.* The WHO public-health approach to antiretroviral treatment against HIV in resource-limited settings. *Lancet* 2006;**368**:505–10. doi:10.1016/S0140-6736(06)69158-7

14 IAS. Differentiated Care for Hiv: a Decision Framework for Antiretroviral Therapy Delivery. *Differ Care.org (International AIDS Soc* 2016;:1–56.

15 WHO. Key considerations for differentiated antiretroviral therapy delivery for specific populations: children, adolescents, pregnant and breastfeeding women and key populations. 2017.

16 International AIDS Society I. Differentiated Service Delivery. IAS. 2019.http://www.differentiatedcare.org/ (accessed 3 Jun 2019).

17 Moore JE, Mascarenhas A, Bain J, *et al.* Developing a comprehensive definition of sustainability. *Implement Sci* 2017;**12**:110. doi:10.1186/s13012-017-0637-1

For peer review only - http://bmjopen.bmj.com/site/about/guidelines.xhtml

18 Whelan J, Love P, Pettman T, *et al.* Cochrane Update Predicting sustainability of intervention effects in public health evidence: identifying key elements to provide guidance. doi:10.1093/pubmed/fdu027
19 Schell SF, Luke DA, Schooley MW, *et al.* Public health program capacity for sustainability: A new framework. *Implement Sci* 2013;**8**:1. doi:10.1186/1748-5908-8-15
20 Gruen RL, Elliott JH, Nolan ML, *et al.* Sustainability science: an integrated approach for health-programme planning. *Lancet* 2008;**372**:1579–89. doi:10.1016/S0140-6736(08)61659-1
21 Hodge LM, Turner KMT. Sustained Implementation of Evidence-based Programs in Disadvantaged Communities: A Conceptual Framework of Supporting Factors. *Am J Community Psychol* 2016;**58**:192–210. doi:10.1002/ajcp.12082
22 Mancini JA, Marek LI. Sustaining Community-Based Programs for Families: Conceptualization and Measurement*. *Fam Relat* 2004;**53**:339–47. doi:10.1111/j.0197-6664.2004.00040.x
23 Sarriot EG, Winch PJ, Ryan LJ, *et al.* A methodological approach and framework for sustainability assessment in NGO-implemented primary health care programs. *Int J Health Plann Manage* 2004;**19**:23–41. doi:10.1002/hpm.744
24 Luke DA, Calhoun A, Robichaux CB, *et al.* The Program Sustainability Assessment Tool: a new instrument for public health programs. *Prev Chronic Dis* 2014;**11**:130184. doi:10.5888/pcd11.130184
25 Lennox L, Maher L, Reed J. Navigating the sustainability landscape: A systematic review of sustainability approaches in healthcare. Implement. Sci. 2018;**13**:27. doi:10.1186/s13012-017-0707-4
26 Callaghan M, Ford N, Schneider H. A systematic review of task- shifting for HIV treatment and care in Africa. Hum. Resour. Health. 2010;**8**:8. doi:10.1186/1478-4491-8-8
27 Philips M, Zachariah R, Venis S. Task shifting for antiretroviral treatment delivery in sub-Saharan Africa: not a panacea. *Lancet* 2008;**371**:682–4. doi:https://dx.doi.org/10.1016/S0140-6736(08)60307-4
28 Kredo T, McCaul M, Volmink J. Task-shifting from doctors to nondoctors for initiation and maintenance of antiretroviral therapy. South African Med. J. 2015;**105**:626–7. doi:10.7196/SAMJnew.8271
29 World Health Organisation (WHO). Hiv, Universal Health Coverage and the Post-2015 Development Agenda. 2015.
30 Fund G. Building Resilient and Sustainable Systems for Health. 2015.
31 Castelli F, Pietra V, Diallo I, *et al.* Antiretroviral (ARV) Therapy in Resource Poor Countries: What do we Need in Real Life? *Open AIDS J* 2010;**4**:28–32. doi:10.2174/1874613601004020028
32 Azeredo TB, Oliveira MA, Santos-Pinto CDB, *et al.* Sustainability of ARV provision in developing countries: challenging a framework based on program history. *Cien Saude Colet* 2017;**22**:2581–94. doi:10.1590/1413-81232017228.29472016
33 Abebe B. Sustainability of HIV/AIDS Care & Support Programmes Acknowledgement-Igratefully acknowledge Prof. Yemane Berhane, program and theory review course. *Type Double Blind Peer Rev Int Res J Publ Glob Journals Inc* 2012;**12**.
34 Ament SMC, De Groot JJA, Maessen JMC, *et al.* Sustainability of professionals' adherence to clinical practice guidelines in medical care: A systematic review. BMJ Open. 2015;**5**:e008073. doi:10.1136/bmjopen-2015-008073
35 Wiltsey Stirman S, Kimberly J, Cook N, *et al.* The sustainability of new programs and innovations: a review of the empirical literature and recommendations for future research. *Implement Sci* 2012;**7**:17. doi:10.1186/1748-5908-7-17
36 Shediac-Rizkallah MC, Bone LR. Planning for the sustainability of community-based health programs: conceptual frameworks and future directions for research, practice and policy. 1998.

For peer review only - http://bmjopen.bmj.com/site/about/guidelines.xhtml

37 Chambers DA, Glasgow RE, Stange KC. The dynamic sustainability framework: addressing the paradox of sustainment amid ongoing change. *Implement Sci* 2013;**8**:1. doi:10.1186/1748-5908-8-117

38 Scheirer MA. Is Sustainability Possible? A Review and Commentary on Empirical Studies of Program Sustainability. *Am J Eval* 2005;**26**:320–47. doi:10.1177/1098214005278752

39 Iwelunmor J, Blackstone S, Veira D, *et al.* Toward the sustainability of health interventions implemented in sub-Saharan Africa: a systematic review and conceptual framework. *Implement Sci* 2016;**11**. doi:10.1186/s13012-016-0392-8

40 Moher D, Shamseer L, Clarke M, *et al.* Preferred reporting items for systematic review and meta-analysis protocols (PRISMA-P) 2015 statement. Published Online First: 2015. doi:10.1186/2046-4053-4-1

41 Ouzzani M, Hammady H, Fedorowicz Z, *et al.* Rayyan-a web and mobile app for systematic reviews. *Syst Rev* 2016;**5**:1–10. doi:10.1186/s13643-016-0384-4

42 Calhoun A, Mainor A, Moreland-Russell S, *et al.* Using the Program Sustainability Assessment Tool to assess and plan for sustainability. *Prev Chronic Dis* 2014;**11**:130185. doi:10.5888/pcd11.130185

43 Popay J, Roberts H, Sowden A, *et al.* Guidance on the Conduct of Narrative Synthesis in Systematic Reviews. *A Prod from ESRC Methods Program* 2006;:211–9. doi:10.1111/j.1523-536x.1995tb00261.x

44 Downs SH, Black N. The feasibility of creating a checklist for the assessment of the methodological quality both of randomised and non-randomised studies of health care interventions. *J Epidemiol Community Heal* 1998;**52**:377–84.

45 Lockwood C, Munn Z, Porritt K. Qualitative research synthesis. *Int J Evid Based Healthc* 2015;**13**:179–87. doi:10.1097/XEB.0000000000000062

46 Guyatt G, Oxman AD, Akl EA, *et al.* GRADE guidelines: 1. Introduction - GRADE evidence profiles and summary of findings tables. *J Clin Epidemiol* 2011;**64**:383–94. doi:10.1016/j.jclinepi.2010.04.026

For peer review only

For peer review only - http://bmjopen.bmj.com/site/about/guidelines.xhtml

Supplementary File I: Search strategy (PubMed & EMBASE)

#1 Pubmed Search ALL <2000 to September 21, 2018>

Search history sorted by search number ascending

#	Searches
1	(patient-centred care or patient-centered care or community supported models or adherence clubs or teen* club or task shifting or ("community ART" adj2 (distribution or delivery or refill)) or community client lead ART-delivery or facility fast track or quick pick-up or (p?ediatric expedited adj2 ART)).mp. or ((differentiated or decentrali?ed or community) adj2 (care or intervention)).ti,ab,kf. or (adherence club or differentiated model* or down-referr* or out-of-clinic).ti,ab,kf.
2	exp HIV/ or exp HIV INFECTIONS/ or exp HIV Seropositivity/ or hiv.ti,ab,kf. or (antiretroviral treatment or ART).ti,ab,kf.
3	1 and 2

#2 Embase search <2000 to 2018 September 21>

Search history sorted by search number ascending

#	Searches
1	(patient-centred care or patient-centered care or community supported models or adherence clubs or teen* club or task shifting or ("community ART" adj2 (distribution or delivery or refill)) or community client lead ART-delivery or facility fast track or quick pick-up or (p?ediatric expedited adj2 ART)).mp. or ((differentiated or decentrali?ed or community) adj2 (care or intervention)).ti,ab,kw. or (adherence club or differentiated model* or down-referr* or out-of-clinic).ti,ab,kw.
2	exp Human immunodeficiency virus/
3	exp Human immunodeficiency virus infection/
4	(hiv or (antiretroviral treatment or ART)).ti,ab,kw.
5	2 or 3 or 4
6	1 and 5
7	limit 6 to (conference abstracts or embase)

For peer review only - http://bmjopen.bmj.com/site/about/guidelines.xhtml

Supplementary File II: Data extraction tool

Publication detail		Setting		Intervention detail																	Study detail		Quantitative Outcomes - any numeric patient-centered outcomes to show effectiveness (*Insert reported outcome values/units/definition only*)																						
Author, Year, Journal	Title of publication	Town, Country e.g. Tete, Mozambique	Setting i.e. Rural or Urban	HIV project/Study site e.g. MSF project	Project ownership i.e. Government (MOH)or NGO (MSF)	Year of commencement of Intervention	Who is the Target population/ Intervention group	Comparison group/contemporary or historical	Definition of "Stable" patient	Number of participants (stable)	Number of participants (comparison)	Model e.g. Decentralized care, DCM (if so which? Task shifting	Brief description of intervention	Level of implementation (number of sites)	Study aim	Study variables definitions	Primary care provider in intervention	Primary care provider in standard of care (SOC)	Provider in intervention funded by	Provider in SOC funded by	Study design	Any Theoretical underpinning for the study	Outcomes - VL (%) SOC	Outcomes - VL (%) DCM intervention	Outcome - VL (rate) SOC	Outcome - VL (rate) DCM intervention	*Comments about units or subgroup analysis*	Outcome - Retention (%)SOC	Outcome - Retention (%)DCM intervention	*Comments about units or any subgroup analysis*	Outcome - LTFU (%)SOC	Outcome - LTFU (%)DCM intervention	Outcome - LTFU (rate) SOC	Outcome - LTFU (rate)	*Comments about units or any subgroup analysis*	Outcome - Mortality (%) SOC	Outcome - Mortality (%) DCM intervention)	Outcome - Mortality (rate)SOC	Outcome - Mortality (rate)DCM intervention	*Comments about units or any subgroup analysis*	Outcome - Provider Cost/visit ($)SOC	Outcome - Provider Cost/visit ($)DCM intervention)	Outcome - Patient. Cost/visit ($)SOC	Outcome - Patient. Cost/visit ($)DCM intervention)	*Comments about units or any subgroup analysis*

Intervention Design and Delivery								External environment				Intervention Process					
**Evidence base** - Is there evidence that the intervention provides the expected benefits as planned i.e. that the DSD improves outcomes	**Expertise** - Is there evidence of adequate expert knowledge and experience to carry out the DSD especially by supporting organisation	**QI methods** - Is there evidence that QI methods i.e. using data to identify gaps which are continually improved, starting with a pilot and then spreading etc. are used to support intervention success and sustainability	**Monitoring progress** - Is there a standardized and systematic method to gather and report data during DSD intervention	**Project duration** - Is there evidence that the intervention will last beyond initial funding	**Project type** - What type of intervention is it e.g. prevention, treatment, palliative, supportive care	**Problem** - Is there general awareness of a problem among stakeholders that requires the DSD intervention to address	**Capacity building** - Is there evidence of any orientation, training, on-going mentoring for staff delivering the DSD intervention	**Awareness** - Is there evidence of the larger community being aware of the DSD intervention and promoting its benefit	**Political support** - Is there evidence that the intervention has political support? e.g. government engagement, guidelines revision to include DSD requirement etc.	**Spread** - Is there evidence that the intervention or underlying concepts spread within participating organisation or to other locations	**Urgency** - Is there evidence of an urgency to maintain the DSD intervention based on its relevance	**Roles & responsibilities** - Is there evidence that roles & responsibilities of staff involved in the DSD are spread out and clearly defined	**Belief in initiative** - Is there evidence that staff think the DSD intervention is a better way to do things	**Complexity** - Is there evidence that it is not difficult for staff to understand and conduct the DSD intervention	**Shared goal** - Is there evidence of a shared aim and vision established with all stakeholders before commencing the DSD intervention	**Incentives** - Is there evidence that rewards or benefits derived from the DSD intervention are considered enough motivation that drive stakeholders to engage and continue delivering intervention over time	**Job requirement /descriptions** - Is there evidence of revision of job requirement for key staff incorporating the DSD intervention tasks as part of key job descriptions
3 = There is sufficient evidence that the intervention provides expected benefits to stakeholders	3 = There is sufficient evidence that the supporting organization has adequate expert knowledge and experience to conduct intervention	3 = There is sufficient evidence of the use of QI methods to support the conduct of intervention	3 = There is sufficient evidence of monitoring the intervention using standardised system to gather and report data over time	3 = There is sufficient evidence the intervention will last for a long time beyond the initial project and funding	3 = The type and design of the project is clear (e.g. preventive, treatment, palliative care, supportive care etc.)	3 = The intervention is addressing a problem that is recognised and accepted as a real concern by all stakeholders i.e. community, staff, patients, supporting organisation, facility, government	3 = There is sufficient evidence of orientation/ training/ mentoring to new staff and on-going training to all staff to be able to deliver intervention successfully	3 = There is sufficient evidence of effort to ensure that stakeholders e.g. the community are aware of the benefits of the intervention through media marketing e.g. patient pressure groups, community leaders	3 = There is sufficient evidence the intervention has the full support of Government (involvement of the MOH, District/State/zonal health unit, institutions, Revision of guideline, inclusion in strategic plans etc.)	3 = There is sufficient evidence that the intervention or beneficial parts of it are spread within a facility or to other facilities in a community or district	3 = There is sufficient evidence of motivation or urgency to maintain the intervention or parts of it based on its perceived potential of supporting a relevant healthcare need.	3 = There is sufficient evidence that roles and responsibilities of all staff involved in the intervention is clear and evenly distributed so no staff is over-burdened	3 = There is sufficient evidence that majority of staff conducting the intervention believe the change is a better way of doing thing and will add value	3 = There is sufficient evidence that it is not hard to understand, conduct and maintain the intervention	3 = There is sufficient evidence of a shared aim and vision for the intervention existing among all major stakeholders including the community, government, partners, patients as well as goal revision when necessary	3 = There is sufficient evidence of perceived benefit from the intervention by all stakeholders including community, supporting organisation, patients, staff and government	3 = There is sufficient evidence of revised job requirement for key staff in facilities which capture the roles and job functions introduced by the intervention e.g. revised job description, SOP, guidelines OR a revision in job requirement was not required
2 = There is some evidence that the intervention provides expected benefits to stakeholders	2 = There is some evidence that the supporting organisation has adequate expert knowledge and experience to conduct intervention	2 = There is some evidence of the use of QI methods to support the conduct of intervention	2 = There is some evidence of monitoring the intervention using standardised system to gather and report data over time	2 = There is some evidence the intervention will last for some time beyond the initial project and funding	2 = The type and design of the project is clear to some extent (e.g. preventive, treatment, palliative care, supportive care etc.)	2 = The intervention is addressing a problem that is recognised and accepted as a concern by some stakeholders i.e. mainly by the facility, patients, staff, supporting organization	2 = There is some evidence of orientation/training/ mentoring to new staff and to all staff to be able to deliver intervention successfully	2 = There is some evidence of effort to ensure that stakeholders e.g. the community are aware of the benefits of the intervention through media marketing e.g. community leaders alone or patients or pressure groups	2 = There is evidence the intervention has some level of support of the Government (involvement of the MOH, District/State/zonal health unit, an institution, etc.)	2 = There is some evidence the intervention spread to a few other sites beyond the intervention facility	2 = There is some evidence of motivation or urgency to maintain the intervention or parts of it based on its perceived potential of supporting a relevant healthcare need.	2 = There is some evidence that roles and responsibilities of staff involved in the intervention is clear and evenly distributed so no staff is over-burdened	2 = There is some evidence that staff conducting the intervention believe the change is a better way of doing thing and will add value	2 = There is evidence of some difficulty in understanding or conducting and maintaining the intervention	2 = There is some evidence of a shared aim and vision for the intervention existing among most stakeholders including the community, a local partners and patients.	2 = There is some evidence of perceived benefit from the intervention by some stakeholders e.g. only supporting organisation or patients or staff	2 = There is some evidence of revised job requirement for key staff in facilities with new roles and job functions introduced by the intervention but no revised job descriptions, SOP
1 = There is little or no evidence that the intervention provides expected benefits to stakeholders	1 = There is little or no evidence the supporting organisation has adequate expert knowledge and experience to conduct intervention	1 = There is little or no evidence of the use of QI methods to support the conduct of intervention to a little or no extent	1 = There is little or no evidence of monitoring the intervention using standardised system to gather and report data over time	1 = There is little or evidence the intervention will last beyond the initial project and funding	1 = The type and design of the project is clear to a little extent (e.g. preventive, treatment, palliative care, supportive care etc.)	1 = The intervention is addressing a problem that is not relly recognised and accepted as a concern by any stakeholders	1 = There is little or no evidence of orientation/training/ mentoring to staff to be able to deliver intervention successfully	1 = Only PLHIV and lay workers in the community participating in the intervention are aware of the intervention	1 = There is evidence the intervention has the support of at least the institution involved in implementation, a local NGO etc.	1 = There is evidence the intervention or beneficial parts of it are spread to at least other parts of a facility	1 = There is little or no evidence of motivation or urgency to maintain the intervention or parts of it based on its perceived potential of supporting a relevant healthcare need.	1 = There is little or no evidence that roles and responsibilities of staff involved in the intervention are clear or evenly distributed	1 = There is little or no evidence that staff conducting the intervention believe the change is a better way of doing things	1 = There is evidence of moderate difficulty in understanding or conducting and maintaining the intervention	1 = There is little or no evidence of a shared aim and vision for the intervention existing among stakeholders. Only the supporting partner developed a goal.	1 = There is little or no evidence of perceived benefit from the intervention by any stakeholder, maybe only the supporting organisation	1 = There is little or no evidence of revised job requirement for the staff invloved with implementing the intervention at the facilities

For peer review only

	Resources				
**Additional workload** - Is there evidence that any additional workload introduced by the DSD intervention is manageable and requiring no special effort to staff involved	**Resources - General** - Is there evidence that resources needed to manage and maintain the DSD intervention are available	**Funding** - Is there evidence that adequate funds are available to implement and strategic funds planned to sustain the DSD intervention	**Infrastructure** - Is there evidence that resources required to support the DSD intervention e.g. office space, materials, and supplies are available	**Staff** - Is there evidence of sufficient staff in place to conduct and sustain the DSD intervention	**Time** - Is there evidence that adequate time was dedicated for the DSD intervention in the routine daily schedule of the facility
3 = There is sufficient evidence that any additional workload introduced by the intervention is manageable and evenly divided among staff without requiring extra effort	3 = There is sufficient evidence that all/most resources required to conduct and maintain the intervention are available and adequate and provided by the government i.e. more government less external donor	3 = There is sufficient evidence that adequate (all/most) funds required to implement and sustain the intervention are available and provided by government	3 = There is sufficient evidence that all/most of the resources required to support the intervention such as buildings, office space, materials and supplies are available and provided by the government	3 = There is sufficient evidence of sufficient number of staff, internal and external (i.e. a team) in place to implement and sustain the intervention employed by the government	3 = There is sufficient evidence that adequate time was dedicated to the intervention activities in the routine daily schedule of the facility
2 = There is evidence that the additional workload introduced by the intervention is manageable to a some extent and evenly divided among staff	2 = There is evidence that some resources required to conduct and maintain the intervention are available and adequate i.e. less government and more external donor	2 = There is evidence that most of the funds required to implement and sustain the intervention are available e.g.less government funds and more external donor funds	2 = There is evidence that the resources required to support the intervention such as buildings, office space, materials and supplies are available i.e. less government support and more external donor support	2 = There is evidence that the number of internal staff in place to implement and sustain the intervention is mostly adequate i.e. the cadre of staff needed is employed by govenrment but paid with external donor funds	2 = There is evidence that time was dedicated to some of the intervention activities in the routine daily schedule of the facility
1 = There is little or no evidence that the additional workload introduced by the intervention is manageable to the staff involved	1 = There is evidence that little or none of the resources required to conduct and maintain the intervention are available and adequate i.e. mostly provided by external donor	1 = There is evidence that little or none of the funds required to implement and sustain the intervention are available e.g.mostly provided by external donor funds	1 = There is evidence that the resources required to support the intervention such as buildings, office space, materials and supplies are available and mostly paid by external funder	1 = There is evidence that the number of internal staff in place to implement and sustain the intervention is somewhat adequate i.e. project staff are mainly employed and paid by extarnal funder	1 = There is evidence that time was dedicated to little or none of the intervention activities in the routine daily schedule of the facility i.e. project activities run within the facility but parallel to routine facility activities

Organizational setting					
**Integration** - Is there evidence that the DSD intervention was embedded within the existing organizational structure, Programme and policies	**Adaptation** - Is there evidence that the DSD intervention is flexible to respond, change, adapt and fit with local context requirement	**Opposition** - Is there evidence of any resistance due to other competing interests from stakeholders reported	**Readiness** - Is there evidence that health facilities have adequate capacity and readiness to undertake the DSD intervention i.e. in terms of materials and manpower	**Organizational Value system and culture** - Is there evidence that the values of the DSD intervention align with health system values, prevailing beliefs and culture and priorities	**Management support** - Is there evidence of facility management support for the delivery and maintenance of the DSD intervention
3 = There is sufficient evidence that the intervention was embedded within the existing organizational structures, programmes and policies of the health system and the facility	3 = There is sufficient evidence that the intervention responds to changes and adapts to fit with local context and requirements	3 = There is sufficient evidence of no resistance from stakeholders to the intervention due to other competing priorities	3 = There is sufficient evidence of adequate capacity and readiness of facilities to conduct the intervention i.e. all materials and staff needed are provided by government	3 = There is sufficient evidence that the health system and facility values, prevailing beliefs and culture and priorities support the sustainability and strategic direction of the intervention e.g. inclusion instragegic plan or guideline	3 = There is sufficient evidence of management total support of the intervention in the form of reminders, staff, technical and education to enhance delivery
2 = There is evidence that the intervention was embedded to some extent within the existing organizational structures, programmes and policies of the facility	2 = There is some evidence that the intervention responds to changes and adapts to fit with local context and requirements	2 = There is evidence of some resistance from stakeholders to the intervention due to other competing priorities	2 = There is some evidence of capacity and readiness of facilities to conduct the intervention i.e. most materials and staff needed are provided by government with support from external funder	2 = There is some evidence that the health system and facility values, prevailing beliefs and culture and priorities support the sustainability and strategic direction of the intervention e.g. some government involvement with little commitment	2 = There is some evidence of management support of the intervention to a large extent in the form of reminders, staff, technical and education to enhance delivery i.e. in principle but it is non-commital
1 = There is evidence that the intervention was embedded to a little extent within the existing organizational structures, programmes and policies of the facility	1 = There is little or no evidence that the intervention responds to changes and adapts to fit with local context and requirements	1 = There is evidence of resistance to a large extent from stakeholders to the intervention	1 = There is evidence that capacity and readiness of facilities to conduct the intervention is limited i.e. aii/most materials and staff needed are provided by external funder	1 = There is little or no evidence that the health system and facility values, prevailing beliefs and culture and priorities support the sustainability and strategic direction of the intervention	1 = There is little or no evidence of management support of the intervention

People Involved								
**Leaders/champions** - Is there evidence of any influential person or group who advocates and supports the DSD intervention	**Ownership** - Is there evidence that stakeholders take ownership to support, embed and sustain the DSD intervention	**Power** - Is there evidence that stakeholders have the ability to use their power to make decisions, advocate and support the DSD intervention	**Collaboration** - Is there evidence of any collaborations, partnerships and support networks to promote and sustain the DSD intervention	**Satisfaction** - Is there evidence of benefits and rewards enjoyed by stakeholders and staff for participation in the DSD intervention reported	**Stakeholders participation** - Is there evidence that key stakeholders (those affected by the intervention) are engaged and participate in the DSD intervention	**Community participation** - Is there evidence of the participation of community members in directing and shaping DSD intervention goals and approaches to reflect their values and needs	**Patient involvement** - Is there evidence of the involvement of patients in DSD intervention processes to understand patient's perspectives, values and needs	**Staff involvement** - Is there evidence of the involvement of staff in the planning, design, delivery of the DSD intervention
3 = There is evidence of an influential person (champion) and group of people (patient pressure group) who have the ability and skills to advocate, communicate and support the intervention e.g. a prominent community leader, a PLHIV group, NGO	3 = There is sufficient evidence that the government, facilities, communities and other stakeholders take ownership and responsibility to support the intervention	3 = There is sufficient evidence of the ability of stakeholders to use their power to make decisions, advocate and support initiative	3 = There is sufficient evidence of partnerships, collaborations and networks to support and sustain the intervention e.g. with government and other local stakeholders, patient groups etc.	3 = There is sufficient evidence of acceptance, enjoyment and reward among stakeholders from participating in intervention e.g. Government, local partners, staff, patients etc.	3 = There is sufficient evidence of the involvement and participation of stakeholders who are affected by the intervention e.g. Government, community, staff, patients	3 = There is sufficient evidence of the participation of community members to direct and shape the intervention to reflect their values, expectations and needs e.g. involving community groups and leaders	3 = There is sufficient evidence of the involvement of patients in the intervention's processes, to understand patient's perspective i.e. in the design and process	3 = There is sufficient evidence of the involvement of staff in the planning, design, delivery and maintenance of the intervention
2 = There is some evidence of an influential person (champion) and group of people (patient pressure group) who have the ability and skills to advocate, communicate and support the intervention e.g. expert patients living openly with their status	2 = There is some evidence that the facilities and other stakeholders i.e. the community or any local partner take ownership and responsibility to support the intervention	2 = There is some evidence that stakeholders have the ability to use their power to make decisions, advocate and support initiative	2 = There is evidence of some level of partnerships, collaborations and networks to support and sustain the intervention e.g. with some local stakeholders	2 = There is evidence of some level of acceptance, enjoyment and reward among stakeholders from participating in intervention e.g. Among direct beneficiaries staff and patients	2 = There is evidence of some level of involvement and participation of stakeholders who are affected by the intervention e.g. staff, patients	2 = There is evidence of some level of participation of community members to direct and shape the intervention to reflect their values, expectations and needs involving community groups e.g. involving lay workers from the community	2 = There is evidence of some level of involvement of patients in the intervention's processes, to understand patient's perspective e.g. adapting the process	2 = There is evidence of some level of involvement of staff in the planning, design, delivery and maintenance of the intervention
1 = There is little or no evidence of an influential patient, or group of people who have the ability and skills to advocate, communicate and support other patients at the facility level during the intervention.	1 = There is little or no evidence that any stakeholder take ownership and responsibility to support the intervention maybe just the facilities	1 = There is little or no evidence of the ability of stakeholders to use their power to make decisions, advocate and support initiative	1 = There is little or no evidence of partnerships, collaborations and networks to support and sustain the intervention e.g. with the facility or institution (s)	1 = There is little or no evidence of acceptance, enjoyment and reward among any stakeholder from participating in intervention	1 = There is little or no evidence of the involvement and participation of stakeholders who are affected by the intervention e.g. patients	1 = There is little or no evidence of participation of community membersto direct and shape the intervention to reflect their values, expectations and needs involving community groups e.g. community only involved as passive recipients	1 = There is little or no evidence of the involvement of patients in the intervention's processes e.g. only as passive participants	1 = There is evidence of involvement of staff in the delivery and maintenance of the intervention

Challenges reported	Comments (Pros & Cons)	Patients perspective	Staff perspective
Any challenges documented during implementation	Any gains or flaws highlighted by staff or patients during implementation	Any experiences or opinions expressed by patients	Any experiences or opinions expressed by staff

For peer review only

Reporting checklist for protocol of a systematic review.

Based on the PRISMA-P guidelines.

Instructions to authors

Complete this checklist by entering the page numbers from your manuscript where readers will find each of the items listed below.

Your article may not currently address all the items on the checklist. Please modify your text to include the missing information. If you are certain that an item does not apply, please write "n/a" and provide a short explanation.

Upload your completed checklist as an extra file when you submit to a journal.

In your methods section, say that you used the PRISMA-Preporting guidelines, and cite them as:

Moher D, Shamseer L, Clarke M, Ghersi D, Liberati A, Petticrew M, Shekelle P, Stewart LA. Preferred Reporting Items for Systematic Review and Meta-Analysis Protocols (PRISMA-P) 2015 statement. Syst Rev. 2015;4(1):1.

		Reporting Item	Page Number
**Title**			
Identification	#1a	Identify the report as a protocol of a systematic review	1
Update	#1b	If the protocol is for an update of a previous systematic review, identify as such	n/a

For peer review only

**Registration**			
	#2	If registered, provide the name of the registry (such as PROSPERO) and registration number	2
**Authors**			
Contact	#3a	Provide name, institutional affiliation, e-mail address of all protocol authors; provide physical mailing address of corresponding author	1
Contribution	#3b	Describe contributions of protocol authors and identify the guarantor of the review	12
**Amendments**			
	#4	If the protocol represents an amendment of a previously completed or published protocol, identify as such and list changes; otherwise, state plan for documenting important protocol amendments	n/a
**Support**			
Sources	#5a	Indicate sources of financial or other support for the review	12
Sponsor	#5b	Provide name for the review funder and / or sponsor	12
Role of sponsor or funder	#5c	Describe roles of funder(s), sponsor(s), and / or institution(s), if any, in developing the protocol	12
**Introduction**			

For peer review only

Rationale	#6	Describe the rationale for the review in the context of what is already known	5
Objectives	#7	Provide an explicit statement of the question(s) the review will address with reference to participants, interventions, comparators, and outcomes (PICO)	6
**Methods**			
Eligibility criteria	#8	Specify the study characteristics (such as PICO, study design, setting, time frame) and report characteristics (such as years considered, language, publication status) to be used as criteria for eligibility for the review	6
Information sources	#9	Describe all intended information sources (such as electronic databases, contact with study authors, trial registers or other grey literature sources) with planned dates of coverage	6
Search strategy	#10	Present draft of search strategy to be used for at least one electronic database, including planned limits, such that it could be repeated	6
Study records - data management	#11a	Describe the mechanism(s) that will be used to manage records and data throughout the review	6
Study records - selection process	#11b	State the process that will be used for selecting studies (such as two independent reviewers) through each phase of the review (that is, screening, eligibility and inclusion in meta-analysis)	7

For peer review only

Study records - data collection process	#11c	Describe planned method of extracting data from reports (such as piloting forms, done independently, in duplicate), any processes for obtaining and confirming data from investigators	7
Data items	#12	List and define all variables for which data will be sought (such as PICO items, funding sources), any pre-planned data assumptions and simplifications	7
Outcomes and prioritization	#13	List and define all outcomes for which data will be sought, including prioritization of main and additional outcomes, with rationale	7-10
Risk of bias in individual studies	#14	Describe anticipated methods for assessing risk of bias of individual studies, including whether this will be done at the outcome or study level, or both; state how this information will be used in data synthesis	11
Data synthesis	#15a	Describe criteria under which study data will be quantitatively synthesised	11
Data synthesis	#15b	If data are appropriate for quantitative synthesis, describe planned summary measures, methods of handling data and methods of combining data from studies, including any planned exploration of consistency (such as I2, Kendall's τ)	11
Data synthesis	#15c	Describe any proposed additional analyses (such as sensitivity or subgroup analyses, meta-regression)	11

For peer review only

Data synthesis	#15d	If quantitative synthesis is not appropriate, describe the type of summary planned	11
Meta-bias(es)	#16	Specify any planned assessment of meta-bias(es) (such as publication bias across studies, selective reporting within studies)	11
Confidence in cumulative evidence	#17	Describe how the strength of the body of evidence will be assessed (such as GRADE)	11

The PRISMA-P checklist is distributed under the terms of the Creative Commons Attribution License CC-BY 4.0. This checklist was completed on 21. July 2019 using https://www.goodreports.org/, a tool made by the EQUATOR Network in collaboration with Penelope.ai

For peer review only

BMJ Open

Evaluating the sustainability of differentiated service delivery interventions for stable ART clients in sub-Saharan Africa: A systematic review protocol

Journal:	*BMJ Open*
Manuscript ID	bmjopen-2019-033156.R1
Article Type:	Protocol
Date Submitted by the Author:	02-Dec-2019
Complete List of Authors:	Okere, Nwanneka; Amsterdam Institute for Global Health and Development, Global Health, University of Amsterdam<br>Urlings, Lisa; Amsterdam University Medical Centres<br>Naniche, Denise; Barcelona Institute for Global Health, University of Barcelona<br>Rinke de Wit, Tobias; Amsterdam Institute for Global Health and Development, Global Health, University of Amsterdam<br>Gomez, Gabriela; London School of Hygiene and Tropical Medicine<br>Hermans, Sabine; Amsterdam Institute for Global Health and Development, Global Health, University of Amsterdam
**Primary Subject Heading**:	HIV/AIDS
Secondary Subject Heading:	Health services research, Public health
Keywords:	Sustainability, Differentiated, Stable, Constructs

SCHOLARONE™
Manuscripts

Evaluating the sustainability of differentiated service delivery interventions for stable ART clients in sub-Saharan Africa: A systematic review protocol

Nwanneka E Okere[1] (0000-0001-9182-6518), Lisa Urlings[2], Denise Naniche[3], Tobias F Rinke de Wit[1], *Gabriela B Gomez[4], *Sabine Hermans[1]

[1] Amsterdam Institute for Global Health and Development, Department of Global Health, Amsterdam UMC, University of Amsterdam, Netherlands
[2] Amsterdam UMC University of Amsterdam Netherlands
[3] Barcelona Institute for Global Health, University of Barcelona, Barcelona, Spain
[4] Department of Global Health and Development London School of Health and Tropical Medicine UK

*These authors share senior authorship

[**Correspondence**: Nwanneka Ebelechukwu Okere (0000-0001-9182-6518), Amsterdam Institute for Global Health and Development AHTC Tower C4 Paasheuvelweg 25 1105 BP Amsterdam, The Netherlands, n.okere@aighd.org, +31 0645991604]

Word count: 2128 excluding title page, abstract, references and tables

Keywords: Sustainability, Differentiated, Constructs, Stable, Funding, Processes

For peer review only

ABSTRACT

Introduction: In 2015, the WHO recommended immediate treatment for people living with HIV (PLHIV). As a result, the number of PLHIV needing antiretroviral therapy (ART) in sub-Saharan Africa (SSA) doubled from 12 million to >25 million. This strain on already weak health systems inspired the exploration of innovative service delivery models so called differentiated service delivery (DSD). DSD tailor services according to client type and offer much-needed improvement in efficiency. The potential of achieving good outcomes for both clients and the health system plus the promise of sustainability motivates DSD promotion especially in low- and medium-income countries (LMIC). This review aims to evaluate the sustainability of DSD interventions.

Methods and analysis: We will systematically review peer-reviewed English literature published between 2000 and 2019 identified by searching PubMed and EMBASE databases. Main inclusion criteria comprise, studies describing DSD interventions conducted in SSA focused on stable adult ART clients and/or compared to clinic-based service delivery. Quality of included studies will be assessed employing the Down & Black's and Joanne Briggs Institute checklists for quantitative and qualitative studies respectively. We will apply a comprehensive sustainability framework including 40 individual constructs to evaluate, score and rank each intervention for sustainability. Narrative and quantitative synthesis and will be conducted as appropriate.

Ethics and dissemination: No ethical approval is required for this study as it is a review of published or publicly available data. Review results will be published in a peer-reviewed journal and presented at international conferences.

PROSPERO registration number: CRD42019120891

For peer review only - http://bmjopen.bmj.com/site/about/guidelines.xhtml

STRENGTHS AND LIMITATIONS

Strengths

1. The current systematic review will assess retrospectively the sustainability of differentiated service delivery models for HIV treatment to date in terms of available evidence on their actual impact, providing essential information for evaluation and implementation.
2. The research question we tackle addresses a topical issue for HIV program especially in SSA. The rapid adoption and expansion of DSD interventions by countries warrants an evaluation of their sustainability. This review will equip policy makers with information necessary for making rational choices to prevent wastage of limited resources.

Limitations

3. A wide range of interventions have been reported and consequently a wide range of outcomes are expected to be included. This could challenge the comparability of sustainability assessments.
4. We propose to review only interventions conducted in SSA and articles published in English, this may prove to bias our review by excluding relevant interventions conducted outside SSA and articles in other languages.

For peer review only

For peer review only - http://bmjopen.bmj.com/site/about/guidelines.xhtml

INTRODUCTION

In 2015, the WHO recommended treatment initiation for people living with HIV (PLHIV), so called 'universal test and treat' strategy. As a result, the number of PLHIV needing antiretroviral treatment (ART) in sub-Saharan Africa (SSA) more than doubled from 12 million to over 25 million [1,2]. This increase in demand adds strain on already weak health systems. It made obvious the insufficiency of traditional clinic-based care and called for innovative service delivery models. Differentiated service delivery (DSD) prioritises client preferences while also aiming to increase health system efficiency [3–8]. DSD has evolved over time and encompasses a wide range of concepts in HIV program implementation especially in low- and medium-income countries (LMIC) such as task shifting, decentralization, community-based ART etc. Ultimately, the goal of DSD is to increase access to and retention in care by reducing demand- and supply-side barriers of clinic-based models of service delivery, especially for rural populations [9–12].

Differentiated care became necessary as more clients were initiated on lifesaving antiretrovirals and ART clinics were becoming crowded. This largely influenced the public health approach involving decentralization recommended by WHO as far back as 2006 [13,14]. Since then, attempts have been made to streamline services by categorizing clients in terms of their clinical profile, such as being stable. Currently, WHO defines a stable client as: "being on ART for at least one year, no current illnesses or pregnancy, a good understanding of lifelong adherence and evidence of treatment success (two consecutive viral load measurements below 1,000 copies/mL)"[1].
Four DSD models were recently described which focus on stable ART clients [15,16]. They include (a) healthcare worker (HCW) groups, (b) facility-based individual models, (c) client managed groups and (d) out-of-facility individual models. Each model is defined by four dimensions, namely 'who' provides care, 'what' is provided, 'when' do the activities happen, and 'where' is care provided. Pre-packaged ART refill, symptom screening, health talks, clinical consultations or referral if unwell defines 'what' care is provided across models. 'When' care is provided as a major benefit of DSD, is usually less frequent than the routine monthly visits for clients categorized as stable. Though WHO recommends 3-6 month intervals, anything between 2-12 month is possible depending on the context.

More distinctively, these models are characterized by the other two dimensions i.e. 'who' provides care (the cadre of healthcare personnel involved) and 'where' care is provided (location). Interventions may be conducted by HCWs within facilities e.g. adherence clubs (ACs), fast track ART refill (FTA), six-monthly appointments (SMA), multi-month scripting (MMS) or by community based HCWs e.g. community drug distribution points (CDDP). Interventions can also be conducted by non-HCWs within facilities e.g. ACs or by community based non-HCWs e.g. Community ART refill groups (CARG), Community ART groups (CAG), Mobile outreach (OR) and AC (see Table 1 - [17]).

For peer review only - http://bmjopen.bmj.com/site/about/guidelines.xhtml

Table 1: Characterization of DSD models

		Location (Where)	
		Within facilities	Community based
Personnel (Who)	HCW-managed	Fast track ART refill (FTR)<br>Six monthly appointment (SMA)<br>Multi-month scripting (MMS)<br>Adherence clubs (AC)<br>(Model 1)	Adherence clubs (AC)<br>Community drug distribution points (CDDP)<br>(Model 2)
	Non-HCW managed	Adherence clubs (AC)<br>(Model 3)	Community ART groups (CAG)<br>Community ART refill groups (CARG)<br>Adherence clubs (AC)<br>Mobile outreach (OR)<br>(Model 4)

The potential of achieving lasting impact and sustainability is the push behind DSD promotion. Sustainability in health care is a broad concept which was only recently comprehensively defined as "program, clinical intervention, and/or implementation strategies which continue to be delivered after a defined period of time (i.e. after expiration of initial external funding) including the maintenance of program or individual behaviour change (i.e., clinician, client) which may evolve or adapt while continuing to produce benefits for individuals/systems" [18]. This definition consolidates key constructs commonly used to describe sustainability of health interventions in literature.

Several conceptual frameworks have been described articulating constructs associated with sustainability of health interventions [19–25]. These frameworks consolidate and elaborate on sustainability as an important concept embedded within the continuum of implementation science [26–28]. Most of them view sustainability as a process to be prospectively explored during implementation rather than an outcome to be retrospectively evaluated. Lennox et al [29], in a recent review, developed a comprehensive framework comprising 40 individual sustainability constructs categorized into six broad domains, namely (1) intervention design and delivery, (2) intervention processes, (3) external environment, (4) organizational setting, (5) resources and (6) people involved.

Why is it important to do this review?

Until a vaccine and/or cure is developed, the long-term sustainability of HIV program interventions is not only desirable but essential, as lifelong ART remains the only option for PLHIV. Therefore, sustainability underlies every strategy so far endorsed for HIV program e.g. task shifting, decentralization of care , differentiation of care [15,30–35]. The increasing call for universal health coverage by the Global Fund to fight AIDS, tuberculosis and malaria and the World Health Organization is similarly motivated and will likely benefit not just HIV program but entire health systems [36,37]. While many DSD interventions have been implemented within HIV program with good outcomes,

For peer review only - http://bmjopen.bmj.com/site/about/guidelines.xhtml

knowledge about their sustainability has been limited [38–40]. It is not clear how these interventions can be properly integrated within the existing health system to ensure they last.

Research on the sustainability of health interventions has been hampered by a lack of consensus. For example, several definitions have existed, some referring to the continuation of an intervention as a whole or in part [41] and others to the continuation of benefits of an intervention [42]. Similarly, several sustainability frameworks exist, making comparability across studies difficult. Common grounds are however beginning to emerge. The framework by Shediac-Rizkallah [43], arguably laid the foundation for identifying commonalities by providing the basic constructs on which other sustainability studies continue to build [44–46]. The recent syntheses of a comprehensive definition and a comprehensive framework, both anchoring on this framework, provided useful tools which we have adopted to gain insight into the sustainability of DSD models in this review [18,29].

In resource constrained settings, the allure of DSD interventions lies not just on the promising client-related outcomes but also the reduced demand on resources it places on health systems without undermining effectiveness. These are thought to make DSD feasible to implement even in LMIC settings. HIV program will therefore require knowledge synthesized from evidence to inform which DSD options to explore. Our review aims to fill this gap by evaluating the sustainability status of available DSD interventions.

METHODS AND ANALYSIS

Protocol registration

This review protocol has been registered in the PROSPERO database (CRD42019120891). The protocol adheres to the Preferred Reporting Items for Systematic Reviews and Meta-Analyses-Protocols (PRISMA-P) 2015 guidelines for preparing protocols of systematic reviews [47].

Search strategy

We will search PubMed and EMBASE to identify eligible articles using a comprehensive search strategy (see Supplementary File I). Additionally, the reference lists of included articles and the reference lists of previously published reviews will be mined for other relevant articles. The review period of interest will be between 2000 and 2019 to capture all articles published since the widespread scale-up of ART services across SSA in the 2000s. Only English language articles will be included. We will use Rayyan QCRI, the free systematic reviews web application [48] to store, organise and manage eligible references whose titles and abstracts will be screened. A follow-up search will be conducted prior to the completion of the review which is planned nine months after commencement.

Eligibility criteria

The inclusion and exclusion criteria we will employ for selecting eligible articles are as listed – see Box 1. Appropriate definitions to guide the selection of eligible articles are also provided – see Box 2.

For peer review only - http://bmjopen.bmj.com/site/about/guidelines.xhtml

Box 1: Eligibility criteria
Inclusions –
- Observational, experimental or quasi-experimental studies.
- Studies involving stable adult ART clients accessing HIV care in SSA.
- Studies describing or assessing HIV services delivered through models other than standard clinic-based care
- Studies which compare the performance of these other service delivery models with standard clinic-based HIV service delivery accessed by other clients. Though, lack of this comparison is not an exclusion criterion.

Exclusions –
- Reviews, editorials, protocol studies and clinical guidelines
- Studies describing or assessing interventions focussed on special population groups e.g. adolescents, children, pregnant women, men who have sex with men, commercial sex workers etc
- Studies utilising data retrospectively collected in electronic databases with little description of the actual intervention

Box 2: Definitions
Stable ART clients
The definition of a stable ART client has been fine-tuned over time, therefore, there will be no restriction in terms of definition. We will rely on what included studies define as stable patients and however with the actual review, provide a summary of definitions employed.

Adult clients
The definition of adult clients varies across studies. We will define adult as ≥18 years but will include studies if the age-range of participants is not < 15 years

Selection of studies
Two reviewers (NEO and UL) will independently screen the titles and abstracts of articles identified in the searches and come up with a list of potential articles. The reviewers will then conduct full-text reading of these articles to determine eligibility. GBG and SMH will be engaged in discussions to make a final determination on eligibility whenever the two reviewers are unable to reach a consensus. Reasons for exclusions will be reported.

Data extraction
We have developed a data extraction form. NEO and UL will pilot the data extraction form on a subset of five articles to assess its functionality and suitability. After incorporating feedback obtained from the review team (NEO, UL, GBG, SMH) a final form will then be used to extract relevant data independently by the two reviewers.

Data to be extracted will include (a) details of each intervention including site, setting, service delivery model, funding, providers, period, theoretical underpinning; (b) study population, participant type and numbers; (c) sustainability constructs as described in Table 2; (d) challenges or un-intended consequences faced implementing the respective DSD, Comments, clients and Staff perspectives (see Supplementary File II).
We speculate that information regarding some constructs in the framework we will be using in our evaluation may be under-reported in the included articles. However, by reporting the extent to which this under-reporting is happening, we hope to highlight this issue for further research. Therefore, we will quantify and report the levels of missing information observed during data extraction (estimated by the number of “not described” assigned by reviewers) per construct for included studies.

For peer review only - http://bmjopen.bmj.com/site/about/guidelines.xhtml

Table 2: Sustainability constructs and evidence to be extracted from included papers (adapted from [29]) (Primary study outcomes)

Domain	Item	Sustainability construct	Outcome	Evaluation Question
**The Intervention Design and Delivery**	1	Demonstrating effectiveness	Patient-related outcomes	Does the paper report any numeric or subjective patient-centred outcomes to show effectiveness e.g. retention-in-care, viral suppression, loss-to-follow-up, patient satisfaction
	2	Evidence base for the intervention	Evidence base	Is there evidence that the intervention provides the expected benefits as planned i.e. that the DSD improves outcomes
	3	Expertise	Expertise	Is there evidence of adequate expert knowledge and experience to carry out the DSD especially by supporting organisation
	4	Quality Improvement (QI)methods	QI methods	Is there evidence that QI methods i.e. using data to identify gaps which are continually improved, starting with a pilot and then spreading etc. are used to support intervention success and sustainability
	5	Monitoring progress	Monitoring progress	Is there a standardized and systematic method to gather and report data during DSD intervention
	6	Intervention duration	Duration	Is there evidence that the intervention will last beyond initial funding
	7	Intervention type	Project Design	What type of intervention is it e.g. prevention, treatment, palliative, supportive care
	8	The problem	Problem awareness	Is there general awareness of a problem among stakeholders that requires the DSD intervention to address
	9	Training & Capacity building	Capacity building	Is there evidence of any orientation, training, on-going mentoring for staff delivering the DSD intervention
**The External Environment**	10	Awareness and Raising the profile	Community awareness	Is there evidence of the larger community being aware of the DSD intervention and promoting its benefit
	11	Socioeconomic and political considerations	Political support	Is there evidence that the intervention has political support? e.g. government engagement, guidelines revision to include DSD requirement etc.
	12	Spread to other organizations	Spread	Is there evidence that the intervention or underlying concepts spread within participating organisation or to other locations
	13	Urgency	Urgency	Is there evidence of an urgency to maintain the intervention based on its relevance
**Intervention processes**	14	Accountability of roles and responsibilities	Roles & responsibilities	Is there evidence that roles & responsibilities of staff involved in the DSD are spread out and clearly defined
	15	Belief in the intervention	Belief in intervention	Is there evidence that staff think the DSD intervention is a better way to do things

For peer review only - http://bmjopen.bmj.com/site/about/guidelines.xhtml

	16	Complexity	Complexity	Is there evidence that it is not difficult for staff to understand and conduct the intervention
	17	Defining Aims and Shared Vision	Shared goal	Is there evidence of a shared aim and vision established with all stakeholders before commencing the intervention
	18	Incentives	Motivation	Is there evidence that rewards, or benefits derived from the DSD intervention are considered enough motivation that drive stakeholders to engage and continue delivering intervention over time
	19	Job requirements	Job requirements	Is there evidence of revision of job requirement for key staff incorporating the DSD intervention tasks as part of key job descriptions
	20	Workload	Workload	Is there evidence that any additional workload introduced by the DSD intervention is manageable and requiring no special effort to staff involved
**Resources**	21	General resources	General resources	Is there evidence that resources needed to manage and maintain the DSD intervention are available
	22	Funding	Funding	Is there evidence that adequate funds are available to implement, and strategic funds planned to sustain intervention i.e. DSD will be embedded and sustained
	23	Infrastructure	Infrastructure	Is there evidence that resources required to support the DSD intervention e.g. office space, materials, and supplies are available
	24	Staff	Staff	Is there evidence of enough staff in place to conduct and sustain DSD intervention
	25	Time	Time	Is there evidence that adequate time was dedicated for DSD intervention in the routine daily schedule of the facility
**Organizational setting**	26	Integration with existing programs and policies	Integration	Is there evidence that DSD intervention was embedded within the existing organizational structure, programs and policies
	27	Intervention adaptation and receptivity	Adaptation	Is there evidence that the DSD intervention is flexible to respond, change, adapt and fit with local context requirement
	28	Opposition	Opposition	Is there evidence of any resistance due to other competing interests from stakeholders reported
	29	Organizational readiness and capacity	Readiness	Is there evidence that health facilities have adequate capacity and readiness to undertake the DSD intervention i.e. in terms of materials and manpower
	30	Organizational values and culture	Values & culture	Is there evidence that the values of the DSD intervention align with health system values, prevailing beliefs and culture and priorities
	31	Support available	Management support	Is there evidence of facility management support for the delivery and maintenance of the DSD intervention

For peer review only - http://bmjopen.bmj.com/site/about/guidelines.xhtml

**The people involved**	32	Leadership and Champions	Champions	Is there evidence of any influential person or group who advocates and supports the DSD intervention
	33	Ownership	Ownership	Is there evidence that stakeholders take ownership to support, embed and sustain the DSD intervention
	34	Power	Power	Is there evidence that stakeholders can use their power to make decisions, advocate and support the DSD intervention
	35	Relationships, collaboration, networks	Collaboration	Is there evidence of any collaborations, partnerships and support networks to promote and sustain the DSD intervention
	36	Satisfaction	Satisfaction	Is there evidence of benefits and rewards enjoyed by stakeholders and staff for participation in DSD intervention reported
	37	Stakeholder participation	Stakeholder participation	Is there evidence that key stakeholders (those affected by the intervention) are engaged and participate in DSD intervention
	38	Community participation	Community participation	Is there evidence of the participation of community members in directing and shaping DSD intervention goals and approaches to reflect their values and needs
	39	Patient involvement	Patient involvement	Is there evidence of the involvement of patients in DSD intervention processes to understand patient's perspectives, values and needs
	40	Staff involvement	Staff involvement	Is there evidence of the involvement of staff in the planning, design, delivery of the DSD intervention

For peer review only - http://bmjopen.bmj.com/site/about/guidelines.xhtml

Data Analysis

Primary outcome measures will include the scores and rankings on sustainability constructs and domains as defined by the Lennox et al framework [29] and presented in Table 2. Since the review aims to extract and score the certainty of evidence retrospectively for sustainability constructs, we simplified a method from a previously described tool to assess sustainability in on-going projects [49]. The two reviewers will assign a score of 3, 2 or 1 independently based on their assessment of whether there was sufficient, some or no evidence about each construct (see Supplementary file II). Additionally, "not described" (ND) will be assigned if the reviewers are not able to make an assessment with information provided. Attempts will be made to contact study authors directly for any additional information or data considered necessary. Secondary outcome measures will include any (a) trends observed in sustainability scores as a result of variations in the criteria employed to define stable clients, (b) if appropriate, any quantitative intervention outcome measures such as retention in care, viral suppression, loss-to-follow-up, client or provider costs etc. reported across a reasonable number of similar interventions, and (c) qualitative outcomes such as perspectives of clients on issues e.g. satisfaction with intervention or HCW, access to ART, peer support) and perspectives of HCWs on issues e.g. workload, care for sick clients. Qualitative outcomes will be extracted as text which will be summarised using narrative synthesis to identify commonly occurring themes across interventions.

Qualitative and quantitative analyses will be conducted. For qualitative analysis, we will conduct narrative synthesis [50] on all included studies. The basic characteristics, key findings including strengths and challenges of DSD interventions will be summarised. For quantitative analysis, we will employ descriptive statistics to determine the sustainability performance of DSD interventions. Performance will be measured in terms of overall construct scores and percentages and median domain scores. This will then be used to rank constructs, domains, interventions and models for sustainability with higher scores ranked as better sustainability.

Quality of evidence

Most published DSD studies are not randomized controlled trials, therefore, NEO and UL will assess risk of bias in included studies using the Downs and Black checklist [51]. The following domains will be assessed: quality of reporting, external validity and internal validity, confounding (selection bias) and power. For qualitative studies, we will assess risk of bias using the Joanna Briggs Institute (JBI) checklist for qualitative research [52]. Disagreements between reviewers will be resolved as for study selection. The overall quality of included studies will be evaluated, if possible, using the Grading of Recommendations Assessment, Development and Evaluation (GRADE) framework for quality of evidence [53].

ETHICS AND DISSEMINATION

No ethical approval is required for the conduct of this systematic review since all data used are freely available in the public space. Results and findings of the review will be shared published in a peer-reviewed journal and presented at appropriate conferences and meetings.

For peer review only - http://bmjopen.bmj.com/site/about/guidelines.xhtml

CONCLUSION

While DSD interventions may not be as onerous as standard clinical care, they pose some demands to the health system. As countries in SSA strive to increase their domestic share of funding for their HIV program, they must be aware of the aspects influencing sustainability of the DSD options they may select. This review will provide information necessary to inform these discussions. Our review also aims to provide the impetus for further enquiry into service delivery models and socio-economic issues necessary to ensure continual client empowerment while supporting minimally resourced health systems.

AUTHORS' CONTRIBUTIONS

NEO, GBG and SH conceived the review design and in refining and registering the review protocol. NEO, GBG, SH and UL contributed to the search strategy. NEO drafted the initial manuscript. NEO, GBG, SH, DN and TRW provided content expertise. All authors contributed to revisions to obtain the final manuscript. All authors read and approved the final manuscript.

PATIENT AND PUBLIC INVOLVEMENT STATEMENT

We will be reviewing and assessing already conducted interventions retrospectively and this will permit little or no involvement of patients and the public.

FUNDING STATEMENT

This work was supported by Erasmus Mundus Joint Doctorate Fellowship, Framework Partnership Agreement 2013-0039, Specific Grant Agreement 2014-0681.

COMPETING INTERESTS' STATEMENT

No competing interests declared

For peer review only - http://bmjopen.bmj.com/site/about/guidelines.xhtml

REFERENCES

1 World Health Organization. Consolidated guidelines on the use of antiretroviral drugs for treating and preventing HIV infection: recommendations for a public health approach. *World Heal Organ* 2016;:155 p. doi:10.1016/j.jped.2014.04.007

2 Pustil R. Global AIDS Update. *Aids* 2016;**17 Suppl 4**:S3-11. doi:10.1073/pnas.86.15.5781

3 Bango F, Ashmore J, Wilkinson L, *et al.* Adherence clubs for long-term provision of antiretroviral therapy: cost-effectiveness and access analysis from Khayelitsha, South Africa. *Trop Med Int Heal* 2016;**21**:1115–23. doi:10.1111/tmi.12736

4 Long L, Brennan A, Fox MP, *et al.* Treatment Outcomes and Cost-Effectiveness of Shifting Management of Stable ART Patients to Nurses in South Africa: An Observational Cohort. *PLoS Med* 2011;**8**:e1001055. doi:10.1371/journal.pmed.1001055

5 Babigumira JB, Castelnuovo B, Stergachis A, *et al.* Cost effectiveness of a Pharmacy-only Refill Program in a large Urban HIV/AIDS clinic in Uganda. *PLoS One* 2011;**6**:e18193. doi:10.1371/journal.pone.0018193

6 Grimsrud A, Bygrave H, Doherty M, *et al.* Reimagining HIV service delivery: The role of differentiated care from prevention to suppression: The. J. Int. AIDS Soc. 2016;**19**. doi:10.7448/IAS.19.1.21484

7 Okoboi S, Ding E, Persuad S, *et al.* Community-based ART distribution system can effectively facilitate long-term program retention and low-rates of death and virologic failure in rural Uganda. *AIDS Res Ther* 2015;**12**. doi:10.1186/s12981-015-0077-4

8 Tsondai PR, Wilkinson LS, Grimsrud A, *et al.* High rates of retention and viral suppression in the scale-up of antiretroviral therapy adherence clubs in Cape Town, South Africa. *J Int AIDS Soc* 2017;**20**. doi:10.7448/IAS.20.5.21649

9 Bedelu M, Ford N, Hilderbrand K, *et al.* Implementing Antiretroviral Therapy in Rural Communities: The Lusikisiki Model of Decentralized HIV/AIDS Care. *J Infect Dis* 2007;**196**:S464–8. doi:10.1086/521114

10 Fredlund VG, Nash J. How Far Should They Walk? Increasing Antiretroviral Therapy Access in a Rural Community in Northern KwaZulu-Natal, South Africa. *J Infect Dis* 2007;**196**:S469–73. doi:10.1086/521115

11 Mutevedzi PC, Lessells RJ, Heller T, *et al.* Scale-up of a decentralized HIV treatment programme in rural KwaZulu-Natal, South Africa: does rapid expansion affect patient outcomes? *Bull World Health Organ* 2010;**88**:593–600. doi:10.2471/BLT.09.069419

12 Davis N, Kanagat N, Sharer M, *et al.* Review of differentiated approaches to antiretroviral therapy distribution. AIDS Care - Psychol. Socio-Medical Asp. AIDS/HIV. 2018;:1–7. doi:10.1080/09540121.2018.1441970

13 WHO Guidelines. *ANTIRETROVIRAL THERAPY FOR HIV INFECTION IN ADULTS AND ADOLESCENTS: Recommendations for a public health approach HIV/AIDS Programme IMPORTANT: ADDENDUM TO 2006 WHO GUIDELINES ON ANTIRETROVIRAL THERAPY FOR HIV INFECTION IN ADULTS AND ADOLESCENTS*. WHO 2006.

14 Gilks CF, Crowley S, Ekpini R, *et al.* The WHO public-health approach to antiretroviral treatment against HIV in resource-limited settings. *Lancet* 2006;**368**:505–10. doi:10.1016/S0140-6736(06)69158-7

15 IAS. Differentiated Care for Hiv: a Decision Framework for Antiretroviral Therapy Delivery. *Differ Care.org (International AIDS Soc* 2016;:1–56.

16 WHO. Key considerations for differentiated antiretroviral therapy delivery for specific populations: children, adolescents, pregnant and breastfeeding women and key populations. 2017.

17 International AIDS Society I. Differentiated Service Delivery. IAS.

For peer review only - http://bmjopen.bmj.com/site/about/guidelines.xhtml

2019.http://www.differentiatedcare.org/ (accessed 3 Jun 2019).

18 Moore JE, Mascarenhas A, Bain J, *et al.* Developing a comprehensive definition of sustainability. *Implement Sci* 2017;**12**:110. doi:10.1186/s13012-017-0637-1

19 Whelan J, Love P, Pettman T, *et al.* Cochrane Update Predicting sustainability of intervention effects in public health evidence: identifying key elements to provide guidance. doi:10.1093/pubmed/fdu027

20 Schell SF, Luke DA, Schooley MW, *et al.* Public health program capacity for sustainability: A new framework. *Implement Sci* 2013;**8**:1. doi:10.1186/1748-5908-8-15

21 Gruen RL, Elliott JH, Nolan ML, *et al.* Sustainability science: an integrated approach for health-programme planning. *Lancet* 2008;**372**:1579–89. doi:10.1016/S0140-6736(08)61659-1

22 Hodge LM, Turner KMT. Sustained Implementation of Evidence-based Programs in Disadvantaged Communities: A Conceptual Framework of Supporting Factors. *Am J Community Psychol* 2016;**58**:192–210. doi:10.1002/ajcp.12082

23 Mancini JA, Marek LI. Sustaining Community-Based Programs for Families: Conceptualization and Measurement*. *Fam Relat* 2004;**53**:339–47. doi:10.1111/j.0197-6664.2004.00040.x

24 Sarriot EG, Winch PJ, Ryan LJ, *et al.* A methodological approach and framework for sustainability assessment in NGO-implemented primary health care programs. *Int J Health Plann Manage* 2004;**19**:23–41. doi:10.1002/hpm.744

25 Luke DA, Calhoun A, Robichaux CB, *et al.* The Program Sustainability Assessment Tool: a new instrument for public health programs. *Prev Chronic Dis* 2014;**11**:130184. doi:10.5888/pcd11.130184

26 Moullin JC, Sabater-Hernández D, Fernandez-Llimos F, *et al.* A systematic review of implementation frameworks of innovations in healthcare and resulting generic implementation framework. *Heal Res Policy Syst* 2015;**13**. doi:10.1186/s12961-015-0005-z

27 Feldstein AC, Glasgow RE. A practical, robust implementation and sustainability model (PRISM) for integrating research findings into practice. *Jt Comm J Qual Patient Saf* 2008;**34**:228–43. doi:10.1016/S1553-7250(08)34030-6

28 Theobald S, Brandes N, Gyapong M, *et al.* Implementation research: new imperatives and opportunities in global health. *Lancet* 2018;**392**:2214–28. doi:10.1016/S0140-6736(18)32205-0

29 Lennox L, Maher L, Reed J. Navigating the sustainability landscape: A systematic review of sustainability approaches in healthcare. Implement. Sci. 2018;**13**:27. doi:10.1186/s13012-017-0707-4

30 Callaghan M, Ford N, Schneider H. A systematic review of task- shifting for HIV treatment and care in Africa. Hum. Resour. Health. 2010;**8**:8. doi:10.1186/1478-4491-8-8

31 Philips M, Zachariah R, Venis S. Task shifting for antiretroviral treatment delivery in sub-Saharan Africa: not a panacea. *Lancet* 2008;**371**:682–4. doi:https://dx.doi.org/10.1016/S0140-6736(08)60307-4

32 Kredo T, McCaul M, Volmink J. Task-shifting from doctors to nondoctors for initiation and maintenance of antiretroviral therapy. South African Med. J. 2015;**105**:626–7. doi:10.7196/SAMJnew.8271

33 Pasipamire L, Nesbitt RC, Ndlovu S, *et al.* Retention on ART and predictors of disengagement from care in several alternative community-centred ART refill models in rural Swaziland. Published Online First: 2018. doi:10.1002/jia2.25183/full

34 Adjetey V, Obiri-Yeboah D, Dornoo B. Differentiated service delivery: A qualitative study of people living with HIV and accessing care in a tertiary facility in Ghana. *BMC Health Serv Res* 2019;**19**. doi:10.1186/s12913-019-3878-7

35 Prust ML, Banda CK, Nyirenda R, *et al.* Multi-month prescriptions, fast-track refills, and community ART groups: results from a process evaluation in Malawi on using differentiated

For peer review only - http://bmjopen.bmj.com/site/about/guidelines.xhtml

models of care to achieve national HIV treatment goals. *J Int AIDS Soc* 2017;**20**:21650. doi:10.7448/IAS.20.5.21650
36 World Health Organisation (WHO). Hiv, Universal Health Coverage and the Post-2015 Development Agenda. 2015.
37 The Global Fund. Building Resilient and Sustainable Systems for Health. 2018.
38 Castelli F, Pietra V, Diallo I, *et al.* Antiretroviral (ARV) Therapy in Resource Poor Countries: What do we Need in Real Life? *Open AIDS J* 2010;**4**:28–32. doi:10.2174/1874613601004020028
39 Azeredo TB, Oliveira MA, Santos-Pinto CDB, *et al.* Sustainability of ARV provision in developing countries: challenging a framework based on program history. *Cien Saude Colet* 2017;**22**:2581–94. doi:10.1590/1413-81232017228.29472016
40 Abebe B. Sustainability of HIV/AIDS Care & Support Programmes Acknowledgement-Igratefully acknowledge Prof. Yemane Berhane, program and theory review course. *Type Double Blind Peer Rev Int Res J Publ Glob Journals Inc* 2012;**12**.
41 Ament SMC, De Groot JJA, Maessen JMC, *et al.* Sustainability of professionals' adherence to clinical practice guidelines in medical care: A systematic review. BMJ Open. 2015;**5**:e008073. doi:10.1136/bmjopen-2015-008073
42 Wiltsey Stirman S, Kimberly J, Cook N, *et al.* The sustainability of new programs and innovations: a review of the empirical literature and recommendations for future research. *Implement Sci* 2012;**7**:17. doi:10.1186/1748-5908-7-17
43 Shediac-Rizkallah MC, Bone LR. Planning for the sustainability of community-based health programs: conceptual frameworks and future directions for research, practice and policy. 1998.
44 Chambers DA, Glasgow RE, Stange KC. The dynamic sustainability framework: addressing the paradox of sustainment amid ongoing change. *Implement Sci* 2013;**8**:1. doi:10.1186/1748-5908-8-117
45 Scheirer MA. Is Sustainability Possible? A Review and Commentary on Empirical Studies of Program Sustainability. *Am J Eval* 2005;**26**:320–47. doi:10.1177/1098214005278752
46 Iwelunmor J, Blackstone S, Veira D, *et al.* Toward the sustainability of health interventions implemented in sub-Saharan Africa: a systematic review and conceptual framework. *Implement Sci* 2016;**11**. doi:10.1186/s13012-016-0392-8
47 Moher D, Shamseer L, Clarke M, *et al.* Preferred reporting items for systematic review and meta-analysis protocols (PRISMA-P) 2015 statement. Published Online First: 2015. doi:10.1186/2046-4053-4-1
48 Ouzzani M, Hammady H, Fedorowicz Z, *et al.* Rayyan-a web and mobile app for systematic reviews. *Syst Rev* 2016;**5**:1–10. doi:10.1186/s13643-016-0384-4
49 Calhoun A, Mainor A, Moreland-Russell S, *et al.* Using the Program Sustainability Assessment Tool to assess and plan for sustainability. *Prev Chronic Dis* 2014;**11**:130185. doi:10.5888/pcd11.130185
50 Popay J, Roberts H, Sowden A, *et al.* Guidance on the Conduct of Narrative Synthesis in Systematic Reviews. *A Prod from ESRC Methods Program* 2006;:211–9. doi:10.1111/j.1523-536x.1995tb00261.x
51 Downs SH, Black N. The feasibility of creating a checklist for the assessment of the methodological quality both of randomised and non-randomised studies of health care interventions. *J Epidemiol Community Heal* 1998;**52**:377–84.
52 Lockwood C, Munn Z, Porritt K. Qualitative research synthesis. *Int J Evid Based Healthc* 2015;**13**:179–87. doi:10.1097/XEB.0000000000000062
53 Guyatt G, Oxman AD, Akl EA, *et al.* GRADE guidelines: 1. Introduction - GRADE evidence profiles and summary of findings tables. *J Clin Epidemiol* 2011;**64**:383–94. doi:10.1016/j.jclinepi.2010.04.026

For peer review only

For peer review only - http://bmjopen.bmj.com/site/about/guidelines.xhtml

For peer review only

For peer review only - http://bmjopen.bmj.com/site/about/guidelines.xhtml

Supplementary File I: Search strategy (PubMed & EMBASE)

#1 Pubmed Search ALL <2000 to November 21, 2019>

Search history sorted by search number ascending

#	Searches
1	(patient-centred care or patient-centered care or community supported models or adherence clubs or teen* club or task shifting or ("community ART" adj2 (distribution or delivery or refill)) or community client lead ART-delivery or facility fast track or quick pick-up or (p?ediatric expedited adj2 ART)).mp. or ((differentiated or decentrali?ed or community) adj2 (care or intervention)).ti,ab,kf. or (adherence club or differentiated model* or down-referr* or out-of-clinic).ti,ab,kf.
2	exp HIV/ or exp HIV INFECTIONS/ or exp HIV Seropositivity/ or hiv.ti,ab,kf. or (antiretroviral treatment or ART).ti,ab,kf.
3	1 and 2

#2 Embase search <2000 to November 21, 2019>

Search history sorted by search number ascending

#	Searches
1	(patient-centred care or patient-centered care or community supported models or adherence clubs or teen* club or task shifting or ("community ART" adj2 (distribution or delivery or refill)) or community client lead ART-delivery or facility fast track or quick pick-up or (p?ediatric expedited adj2 ART)).mp. or ((differentiated or decentrali?ed or community) adj2 (care or intervention)).ti,ab,kw. or (adherence club or differentiated model* or down-referr* or out-of-clinic).ti,ab,kw.
2	exp Human immunodeficiency virus/
3	exp Human immunodeficiency virus infection/
4	(hiv or (antiretroviral treatment or ART)).ti,ab,kw.
5	2 or 3 or 4
6	1 and 5
7	limit 6 to (conference abstracts or embase)

Supplementary File II: Data extraction tool

Publication detail		Setting		Intervention detail																	Study detail	
Author, Year, Journal	Title of publication	Town, Country e.g. Tete, Mozambique	Setting i.e. Rural or Urban	HIV project/Study site e.g. MSF project	Project ownership i.e. Government (MOH)or NGO (MSF)	Year of commencement of Intervention	Who is the Target population/ Intervention group	Comparison group/contemporary or historical	Definition of "Stable" patient	Number of participants (stable)	Number of participants (comparison)	Model e.g. Decentralized care, DCM (if so which? Task shifting	Brief description of intervention	Level of implementation (number of sites)	Study aim	Study variables definitions	Primary care provider in intervention	Primary care provider in standard of care (SOC)	Provider in intervention funded by	Provider in SOC funded by	Study design	Any Theoretical underpinning for the study

**Quantitative Outcomes** - any numeric patient-centered outcomes to show effectiveness (***Insert reported outcome values/units/definition only***)																						
Outcomes - VL (%) SOC	Outcomes - VL (%) DCM intervention	Outcome - VL (rate) SOC	Outcome - VL (rate) DCM intervention	*Comments about units or any subgroup analysis*	Outcome - Retention (%)SOC	Outcome - Retention (%)DCM intervention	*Comments about units or any subgroup analysis*	Outcome - LTFU (%)SOC	Outcome - LTFU (%)DCM intervention	Outcome - LTFU (rate) SOC	Outcome - LTFU (rate)	*Comments about units or any subgroup analysis*	Outcome - Mortality (%) SOC	Outcome - Mortality (%) DCM intervention)	Outcome - Mortality (rate)SOC	Outcome - Mortality (rate)DCM intervention	*Comments about units or any subgroup analysis*	Outcome - Provider Cost/visit ($)SOC	Outcome - Provider Cost/visit ($)DCM intervention)	Outcome - Patient. Cost/visit ($)SOC	Outcome - Patient. Cost/visit ($)DCM intervention)	*Comments about units or any subgroup analysis*

Intervention Design and Delivery							
**Evidence base** - Is there evidence that the intervention provides the expected benefits as planned i.e. that the DSD improves outcomes	**Expertise** - Is there evidence of adequate expert knowledge and experience to carry out the DSD especially by supporting organisation	**QI methods** - Is there evidence that QI methods i.e. using data to identify gaps which are continually improved, starting with a pilot and then spreading etc. are used to support intervention success and sustainability	**Monitoring progress** - Is there a standardized and systematic method to gather and report data during DSD intervention	**Project duration** - Is there evidence that the intervention will last beyond initial funding	**Project type** - What type of intervention is it e.g. prevention, treatment, palliative, supportive care	**Problem** - Is there general awareness of a problem among stakeholders that requires the DSD intervention to address	**Capacity building** - Is there evidence of any orientation, training, on-going mentoring for staff delivering the DSD intervention
3 = There is sufficient evidence that the intervention provides expected benefits to stakeholders	3 = There is sufficient evidence that the supporting organization has adequate expert knowledge and experience to conduct intervention	3 = There is sufficient evidence of the use of QI methods to support the conduct of intervention	3 = There is sufficient evidence of monitoring the intervention using standardised system to gather and report data over time	3 = There is sufficient evidence the intervention will last for a long time beyond the initial project and funding	3 = The type and design of the project is clear (e.g. preventive, treatment, palliative care, supportive care etc.)	3 = The intervention is addressing a problem that is recognised and accepted as a real concern by all stakeholders i.e. community, staff, patients, supporting organisation, facility, government	3 = There is sufficient evidence of orientation/ training/ mentoring to new staff and on-going training to all staff to be able to deliver intervention successfully
2 = There is some evidence that the intervention provides expected benefits to stakeholders	2 = There is some evidence that the supporting organisation has adequate expert knowledge and experience to conduct intervention	2 = There is some evidence of the use of QI methods to support the conduct of intervention	2 = There is some evidence of monitoring the intervention using standardised system to gather and report data over time	2 = There is some evidence the intervention will last for some time beyond the initial project and funding	2 = The type and design of the project is clear to some extent (e.g. preventive, treatment, palliative care, supportive care etc.)	2 = The intervention is addressing a problem that is recognised and accepted as a concern by some stakeholders i.e. mainly by the facility, patients, staff, supporting organization	2 = There is some evidence of orientation/training/ mentoring to new staff and to all staff to be able to deliver intervention successfully
1 = There is little or no evidence that the intervention provides expected benefits to stakeholders	1 = There is little or no evidence the supporting organisation has adequate expert knowledge and experience to conduct intervention	1 = There is little or no evidence of the use of QI methods to support the conduct of intervention to a little or no extent	1 = There is little or no evidence of monitoring the intervention using standardised system to gather and report data over time	1 = There is little or evidence the intervention will last beyond the initial project and funding	1 = The type and design of the project is clear to a little extent (e.g. preventive, treatment, palliative care, supportive care etc.)	1 = The intervention is addressing a problem that is not relly recognised and accepted as a concern by any stakeholders	1 = There is little or no evidence of orientation/training/ mentoring to staff to be able to deliver intervention successfully

External environment			
**Awareness** - Is there evidence of the larger community being aware of the DSD intervention and promoting its benefit	**Political support** - Is there evidence that the intervention has political support? e.g. government engagement, guidelines revision to include DSD requirement etc.	**Spread** - Is there evidence that the intervention or underlying concepts spread within participating organisation or to other locations	**Urgency** - Is there evidence of an urgency to maintain the DSD intervention based on its relevance
3 = There is sufficient evidence of effort to ensure that stakeholders e.g. the community are aware of the benefits of the intervention through media marketing e.g. patient pressure groups, community leaders	3 = There is sufficient evidence the intervention has the full support of Government (involvement of the MOH, District/State/zonal health unit, institutions, Revision of guideline, inclusion in strategic plans etc.)	3 = There is sufficient evidence that the intervention or beneficial parts of it are spread within a facility or to other facilities in a community or district	3 = There is sufficient evidence of motivation or urgency to maintain the intervention or parts of it based on its perceived potential of supporting a relevant healthcare need.
2 = There is some evidence of effort to ensure that stakeholders e.g. the community are aware of the benefits of the intervention through media marketing e.g. community leaders alone or patients or pressure groups	2 = There is evidence the intervention has some level of support of the Government (involvement of the MOH, District/State/zonal health unit, an institution, etc.)	2 = There is some evidence the intervention spread to a few other sites beyond the intervention facility	2 = There is some evidence of motivation or urgency to maintain the intervention or parts of it based on its perceived potential of supporting a relevant healthcare need.
1 = Only PLHIV and lay workers in the community participating in the intervention are aware of the intervention	1 = There is evidence the intervention has the support of at least the institution involved in implementation, a local NGO etc.	1 = There is evidence the intervention or beneficial parts of it are spread to at least other parts of a facility	1 = There is little or no evidence of motivation or urgency to maintain the intervention or parts of it based on its perceived potential of supporting a relevant healthcare need.

Intervention Process					
**Roles & responsibilities** - Is there evidence that roles & responsibilities of staff involved in the DSD are spread out and clearly defined	**Belief in initiative** - Is there evidence that staff think the DSD intervention is a better way to do things	**Complexity** - Is there evidence that it is not difficult for staff to understand and conduct the DSD intervention	**Shared goal** - Is there evidence of a shared aim and vision established with all stakeholders before commencing the DSD intervention	**Incentives** - Is there evidence that rewards or benefits derived from the DSD intervention are considered enough motivation that drive stakeholders to engage and continue delivering intervention over time	**Job requirement /descriptions** - Is there evidence of revision of job requirement for key staff incorporating the DSD intervention tasks as part of key job descriptions
3 = There is sufficient evidence that roles and responsibilities of all staff involved in the intervention is clear and evenly distributed so no staff is over-burdened	3 = There is sufficient evidence that majority of staff conducting the intervention believe the change is a better way of doing thing and will add value	3 = There is sufficient evidence that it is not hard to understand, conduct and maintain the intervention	3 = There is sufficient evidence of a shared aim and vision for the intervention existing among all major stakeholders including the community, government, partners, patients as well as goal revision when necessary	3 = There is sufficient evidence of perceived benefit from the intervention by all stakeholders including community, supporting organisation, patients, staff and government	3 = There is sufficient evidence of revised job requirement for key staff in facilities which capture the roles and job functions introduced by the intervention e.g. revised job description, SOP, guidelines OR a revision in job requirement was not required
2 = There is some evidence that roles and responsibilities of staff involved in the intervention is clear and evenly distributed so no staff is over-burdened	2 = There is some evidence that staff conducting the intervention believe the change is a better way of doing thing and will add value	2 = There is evidence of some difficulty in understanding or conducting and maintaining the intervention	2 = There is some evidence of a shared aim and vision for the intervention existing among most stakeholders including the community, a local partners and patients.	2 = There is some evidence of perceived benefit from the intervention by some stakeholders e.g. only supporting organisation or patients or staff	2 = There is some evidence of revised job requirement for key staff in facilities with new roles and job functions introduced by the intervention but no revised job descriptions, SOP
1 = There is little or no evidence that roles and responsibilities of staff involved in the intervention are clear or evenly distributed	1 = There is little or no evidence that staff conducting the intervention believe the change is a better way of doing things	1 = There is evidence of moderate difficulty in understanding or conducting and maintaining the intervention	1 = There is little or no evidence of a shared aim and vision for the intervention existing among stakeholders. Only the supporting partner developed a goal.	1 = There is little or no evidence of perceived benefit from the intervention by any stakeholder, maybe only the supporting organisation	1 = There is little or no evidence of revised job requirement for the staff invloved with implementing the intervention at the facilities

For peer review only

	Resources					Organizational setting						People Involved												
**Additional workload** - Is there evidence that any additional workload introduced by the DSD intervention is manageable and requiring no special effort to staff involved	**Resources - General** - Is there evidence that resources needed to manage and maintain the DSD intervention are available	**Funding** - Is there evidence that adequate funds are available to implement and strategic funds planned to sustain the DSD intervention	**Infrastructure** - Is there evidence that resources required to support the DSD intervention e.g. office space, materials, and supplies are available	**Staff** - Is there evidence of sufficient staff in place to conduct and sustain the DSD intervention	**Time** - Is there evidence that adequate time was dedicated for the DSD intervention in the routine daily schedule of the facility	**Integration** - Is there evidence that the DSD intervention was embedded within the existing organizational structure, Programme and policies	**Adaptation** - Is there evidence that the DSD intervention is flexible to respond, change, adapt and fit with local context requirement	**Opposition** - Is there evidence of any resistance due to other competing interests from stakeholders reported	**Readiness** - Is there evidence that health facilities have adequate capacity and readiness to undertake the DSD intervention i.e. in terms of materials and manpower	**Organizational Value system and culture** - Is there evidence that the values of the DSD intervention align with health system values, prevailing beliefs and culture and priorities	**Management support** - Is there evidence of facility management support for the delivery and maintenance of the DSD intervention	**Leaders/champions** - Is there evidence of any influential person or group who advocates and supports the DSD intervention	**Ownership** - Is there evidence that stakeholders take ownership to support, embed and sustain the DSD intervention	**Power** - Is there evidence that stakeholders have the ability to use their power to make decisions, advocate and support the DSD intervention	**Collaboration** - Is there evidence of any collaborations, partnerships and support networks to promote and sustain the DSD intervention	**Satisfaction** - Is there evidence of benefits and rewards enjoyed by stakeholders and staff for participation in the DSD intervention reported	**Stakeholders participation** - Is there evidence that key stakeholders (those affected by the intervention) are engaged and participate in the DSD intervention	**Community participation** - Is there evidence of the participation of community members in directing and shaping DSD intervention goals and approaches to reflect their values and needs	**Patient involvement** - Is there evidence of the involvement of patients in DSD intervention processes to understand patient's perspectives, values and needs	**Staff involvement** - Is there evidence of the involvement of staff in the planning, design, delivery of the DSD intervention	Challenges reported	Comments (Pros & Cons)	Patients perspective	Staff perspective
3 = There is sufficient evidence that any additional workload introduced by the intervention is manageable and evenly divided among staff without requiring extra effort	3 = There is sufficient evidence that all/most resources required to conduct and maintain the intervention are available and adequate and provided by the government i.e. more government less external donor	3 = There is sufficient evidence that adequate (all/most) funds required to implement and sustain the intervention are available and provided by government	3 = There is sufficient evidence that all/most of the resources required to support the intervention such as buildings, office space, materials and supplies are available and provided by the government	3 = There is sufficient evidence of sufficient number of staff, internal and external (i.e. a team) in place to implement and sustain the intervention employed by the government	3 = There is sufficient evidence that adequate time was dedicated to the intervention activities in the routine daily schedule of the facility	3 = There is sufficient evidence that the intervention was embedded within the existing organizational structures, programmes and policies of the health system and the facility	3 = There is sufficient evidence that the intervention responds to changes and adapts to fit with local context and requirements	3 = There is sufficient evidence of no resistance from stakeholders to the intervention due to other competing priorities	3 = There is sufficient evidence of adequate capacity and readiness of facilities to conduct the intervention i.e. all materials and staff needed are provided by government	3 = There is sufficient evidence that the health system and facility values, prevailing beliefs and culture and priorities support the sustainability and strategic direction of the intervention e.g. inclusion instragegic plan or guideline	3 = There is sufficient evidence of management total support of the intervention in the form of reminders, staff, technical and education to enhance delivery	3 = There is evidence of an influential person (champion) and group of people (patient pressure group) who have the ability and skills to advocate, communicate and support the intervention e.g. a prominent community leader, a PLHIV group, NGO	3 = There is sufficient evidence that the government, facilities, communities and other stakeholders take ownership and responsibility to support the intervention	3 = There is sufficient evidence of the ability of stakeholders to use their power to make decisions, advocate and support initiative	3 = There is sufficient evidence of partnerships, collaborations and networks to support and sustain the intervention e.g. with government and other local stakeholders, patient groups etc.	3 = There is sufficient evidence of acceptance, enjoyment and reward among stakeholders from participating in intervention e.g. Government, local partners, staff, patients etc.	3 = There is sufficient evidence of the involvement and participation of stakeholders who are affected by the intervention e.g. Government, community, staff, patients	3 = There is sufficient evidence of the participation of community members to direct and shape the intervention to reflect their values, expectations and needs e.g. involving community groups and leaders	3 = There is sufficient evidence of the involvement of patients in the intervention's processes, to understand patient's perspective i.e. in the design and process	3 = There is sufficient evidence of the involvement of staff in the planning, design, delivery and maintenance of the intervention	Any challenges documented during implementation	Any gains or flaws highlighted by staff or patients during implementation	Any experiences or opinions expressed by patients	Any experiences or opinions expressed by staff
2 = There is evidence that the additional workload introduced by the intervention is manageable to a some extent and evenly divided among staff	2 = There is evidence that some resources required to conduct and maintain the intervention are available and adequate i.e. less government and more external donor	2 = There is evidence that most of the funds required to implement and sustain the intervention are available e.g.less government funds and more external donor funds	2 = There is evidence that the resources required to support the intervention such as buildings, office space, materials and supplies are available i.e. less government support and more external donor support	2 = There is evidence that the number of internal staff in place to implement and sustain the intervention is mostly adequate i.e. the cadre of staff needed is employed by govenrment but paid with external donor funds	2 = There is evidence that time was dedicated to some of the intervention activities in the routine daily schedule of the facility	2 = There is evidence that the intervention was embedded to some extent within the existing organizational structures, programmes and policies of the facility	2 = There is some evidence that the intervention responds to changes and adapts to fit with local context and requirements	2 = There is evidence of some resistance from stakeholders to the intervention due to other competing priorities	2 = There is some evidence of capacity and readiness of facilities to conduct the intervention i.e. most materials and staff needed are provided by government with support from external funder	2 = There is some evidence that the health system and facility values, prevailing beliefs and culture and priorities support the sustainability and strategic direction of the intervention e.g. some government involvement with little commitment	2 = There is some evidence of management support of the intervention to a large extent in the form of reminders, staff, technical and education to enhance delivery i.e. in principle but it is non-commital	2 = There is some evidence of an influential person (champion) and group of people (patient pressure group) who have the ability and skills to advocate, communicate and support the intervention e.g. expert patients living openly with their status	2 = There is some evidence that the facilities and other stakeholders i.e. the community or any local partner take ownership and responsibility to support the intervention	2 = There is some evidence that stakeholders have the ability to use their power to make decisions, advocate and support initiative	2 = There is evidence of some level of partnerships, collaborations and networks to support and sustain the intervention e.g. with some local stakeholders	2 = There is evidence of some level of acceptance, enjoyment and reward among stakeholders from participating in intervention e.g. Among direct beneficiaries staff and patients	2 = There is evidence of some level of involvement and participation of stakeholders who are affected by the intervention e.g. staff, patients	2 = There is evidence of some level of participation of community members to direct and shape the intervention to reflect their values, expectations and needs involving community groups e.g. involving lay workers from the community	2 = There is evidence of some level of involvement of patients in the intervention's processes, to understand patient's perspective e.g. adapting the process	2 = There is evidence of some level of involvement of staff in the planning, design, delivery and maintenance of the intervention				
1 = There is little or no evidence that the additional workload introduced by the intervention is manageable to the staff involved	1 = There is evidence that little or none of the resources required to conduct and maintain the intervention are available and adequate i.e. mostly provided by external donor	1 = There is evidence that little or none of the funds required to implement and sustain the intervention are available e.g.mostly provided by external donor funds	1 = There is evidence that the resources required to support the intervention such as buildings, office space, materials and supplies are available and mostly paid by external funder	1 = There is evidence that the number of internal staff in place to implement and sustain the intervention is somewhat adequate i.e. project staff are mainly employed and paid by extarnal funder	1 = There is evidence that time was dedicated to little or none of the intervention activities in the routine daily schedule of the facility i.e. project activities run within the facility but parallel to routine facility activities	1 = There is evidence that the intervention was embedded to a little extent within the existing organizational structures, programmes and policies of the facility	1 = There is little or no evidence that the intervention responds to changes and adapts to fit with local context and requirements	1 = There is evidence of resistance to a large extent from stakeholders to the intervention	1 = There is evidence that capacity and readiness of facilities to conduct the intervention is limited i.e. aii/most materials and staff needed are provided by external funder	1 = There is little or no evidence that the health system and facility values, prevailing beliefs and culture and priorities support the sustainability and strategic direction of the intervention	1 = There is little or no evidence of management support of the intervention	1 = There is little or no evidence of an influential patient, or group of people who have the ability and skills to advocate, communicate and support other patients at the facility level during the intervention.	1 = There is little or no evidence that any stakeholder take ownership and responsibility to support the intervention maybe just the facilities	1 = There is little or no evidence of the ability of stakeholders to use their power to make decisions, advocate and support initiative	1 = There is little or no evidence of partnerships, collaborations and networks to support and sustain the intervention e.g. with the facility or institution (s)	1 = There is little or no evidence of acceptance, enjoyment and reward among any stakeholder from participating in intervention	1 = There is little or no evidence of the involvement and participation of stakeholders who are affected by the intervention e.g. patients	1 = There is little or no evidence of participation of community memberst o direct and shape the intervention to reflect their values, expectations and needs involving community groups e.g. community only involved as passive recipients	1 = There is little or no evidence of the involvement of patients in the intervention's processes e.g. only as passive participants	1 = There is evidence of involvement of staff in the delivery and maintenance of the intervention				

For peer review only

Reporting checklist for protocol of a systematic review.

Based on the PRISMA-P guidelines.

Instructions to authors

Complete this checklist by entering the page numbers from your manuscript where readers will find each of the items listed below.

Your article may not currently address all the items on the checklist. Please modify your text to include the missing information. If you are certain that an item does not apply, please write "n/a" and provide a short explanation.

Upload your completed checklist as an extra file when you submit to a journal.

In your methods section, say that you used the PRISMA-Preporting guidelines, and cite them as:

Moher D, Shamseer L, Clarke M, Ghersi D, Liberati A, Petticrew M, Shekelle P, Stewart LA. Preferred Reporting Items for Systematic Review and Meta-Analysis Protocols (PRISMA-P) 2015 statement. Syst Rev. 2015;4(1):1.

For peer review only

		Reporting Item	Page Number
**Title**			
Identification	#1a	Identify the report as a protocol of a systematic review	1
Update	#1b	If the protocol is for an update of a previous systematic review, identify as such	n/a

**Registration**			
	#2	If registered, provide the name of the registry (such as PROSPERO) and registration number	2
**Authors**			
Contact	#3a	Provide name, institutional affiliation, e-mail address of all protocol authors; provide physical mailing address of corresponding author	1
Contribution	#3b	Describe contributions of protocol authors and identify the guarantor of the review	12
**Amendments**			
	#4	If the protocol represents an amendment of a previously completed or published protocol, identify as such and list changes; otherwise, state plan for documenting important protocol amendments	n/a
**Support**			
Sources	#5a	Indicate sources of financial or other support for the review	12
Sponsor	#5b	Provide name for the review funder and / or sponsor	12
Role of sponsor or funder	#5c	Describe roles of funder(s), sponsor(s), and / or institution(s), if any, in developing the protocol	12
**Introduction**			

For peer review only

Rationale	#6	Describe the rationale for the review in the context of what is already known	5
Objectives	#7	Provide an explicit statement of the question(s) the review will address with reference to participants, interventions, comparators, and outcomes (PICO)	6
**Methods**			
Eligibility criteria	#8	Specify the study characteristics (such as PICO, study design, setting, time frame) and report characteristics (such as years considered, language, publication status) to be used as criteria for eligibility for the review	7
Information sources	#9	Describe all intended information sources (such as electronic databases, contact with study authors, trial registers or other grey literature sources) with planned dates of coverage	6
Search strategy	#10	Present draft of search strategy to be used for at least one electronic database, including planned limits, such that it could be repeated	6
Study records - data management	#11a	Describe the mechanism(s) that will be used to manage records and data throughout the review	6
Study records - selection process	#11b	State the process that will be used for selecting studies (such as two independent reviewers) through each phase of the review (that is, screening, eligibility and inclusion in meta-analysis)	7

For peer review only

Study records - data collection process	#11c	Describe planned method of extracting data from reports (such as piloting forms, done independently, in duplicate), any processes for obtaining and confirming data from investigators	7
Data items	#12	List and define all variables for which data will be sought (such as PICO items, funding sources), any pre-planned data assumptions and simplifications	7
Outcomes and prioritization	#13	List and define all outcomes for which data will be sought, including prioritization of main and additional outcomes, with rationale	8-10
Risk of bias in individual studies	#14	Describe anticipated methods for assessing risk of bias of individual studies, including whether this will be done at the outcome or study level, or both; state how this information will be used in data synthesis	11
Data synthesis	#15a	Describe criteria under which study data will be quantitatively synthesised	11
Data synthesis	#15b	If data are appropriate for quantitative synthesis, describe planned summary measures, methods of handling data and methods of combining data from studies, including any planned exploration of consistency (such as I2, Kendall's τ)	11
Data synthesis	#15c	Describe any proposed additional analyses (such as sensitivity or subgroup analyses, meta-regression)	11

For peer review only

Data synthesis	#15d	If quantitative synthesis is not appropriate, describe the type of summary planned	11
Meta-bias(es)	#16	Specify any planned assessment of meta-bias(es) (such as publication bias across studies, selective reporting within studies)	11
Confidence in cumulative evidence	#17	Describe how the strength of the body of evidence will be assessed (such as GRADE)	11

The PRISMA-P checklist is distributed under the terms of the Creative Commons Attribution License CC-BY 4.0. This checklist was completed on 21. July 2019 using https://www.goodreports.org/, a tool made by the EQUATOR Network in collaboration with Penelope.ai

For peer review only

Evaluating the sustainability of differentiated service delivery interventions for stable ART clients in sub-Saharan Africa: A systematic review protocol

Journal:	*BMJ Open*
Manuscript ID	bmjopen-2019-033156.R2
Article Type:	Protocol
Date Submitted by the Author:	18-Dec-2019
Complete List of Authors:	Okere, Nwanneka; Amsterdam Institute for Global Health and Development, Global Health, University of Amsterdam<br>Urlings, Lisa; Amsterdam University Medical Centres<br>Naniche, Denise; Barcelona Institute for Global Health, University of Barcelona<br>Rinke de Wit, Tobias; Amsterdam Institute for Global Health and Development, Global Health, University of Amsterdam<br>Gomez, Gabriela; London School of Hygiene and Tropical Medicine<br>Hermans, Sabine; Amsterdam Institute for Global Health and Development, Global Health, University of Amsterdam
**Primary Subject Heading**:	HIV/AIDS
Secondary Subject Heading:	Health services research, Public health
Keywords:	Sustainability, Differentiated, Stable, Constructs

SCHOLARONE™ Manuscripts

Evaluating the sustainability of differentiated service delivery interventions for stable ART clients in sub-Saharan Africa: A systematic review protocol

Nwanneka E Okere[1] (0000-0001-9182-6518), Lisa Urlings[2], Denise Naniche[3], Tobias F Rinke de Wit[1], *Gabriela B Gomez[4], *Sabine Hermans[1]

[1] Amsterdam Institute for Global Health and Development, Department of Global Health, Amsterdam UMC, University of Amsterdam, Netherlands
[2] Amsterdam UMC University of Amsterdam Netherlands
[3] Barcelona Institute for Global Health, University of Barcelona, Barcelona, Spain
[4] Department of Global Health and Development London School of Health and Tropical Medicine UK

*These authors share senior authorship

[**Correspondence**: Nwanneka Ebelechukwu Okere (0000-0001-9182-6518), Amsterdam Institute for Global Health and Development AHTC Tower C4 Paasheuvelweg 25 1105 BP Amsterdam, The Netherlands, n.okere@aighd.org, +31 0645991604]

Word count: 2128 excluding title page, abstract, references and tables

Keywords: Sustainability, Differentiated care, Constructs, Stable patients, Processes

For peer review only

For peer review only - http://bmjopen.bmj.com/site/about/guidelines.xhtml

ABSTRACT

Introduction: In 2015, the World Health Organisation (WHO) recommended immediate treatment for people living with HIV (PLHIV). As a result, the number of PLHIV needing antiretroviral therapy (ART) in sub-Saharan Africa (SSA) doubled from 12 million to over 25 million. This put a strain on already weak health systems and inspired the exploration of innovative service delivery models - differentiated service delivery (DSD). In DSD, services are tailored according to client clinical type and offer much-needed improvement in efficiency. The potential of achieving good outcomes for both clients and the health system plus the promise of sustainability motivates DSD promotion especially in low- and medium-income countries (LMIC). This review aims to evaluate the sustainability of DSD interventions.

Methods and analysis: We will systematically review peer-reviewed English literature published between 2000 and 2019 identified by searching PubMed and EMBASE databases. Main inclusion criteria comprise studies describing DSD interventions conducted in SSA focused on stable adult ART clients, whether described alone or compared to clinic-based service delivery. Quality of included studies will be assessed employing the Down & Black's and Joanne Briggs Institute checklists for quantitative and qualitative studies respectively. We will apply a comprehensive sustainability framework including 40 individual constructs to evaluate, score and rank each intervention for sustainability. Narrative and quantitative synthesis will be conducted as appropriate.

Ethics and dissemination: No ethical approval is required for this study as it is a review of published or publicly available data. Review results will be published in a peer-reviewed journal and presented at international conferences.

PROSPERO registration number: CRD42019120891

For peer review only

For peer review only - http://bmjopen.bmj.com/site/about/guidelines.xhtml

STRENGTHS AND LIMITATIONS

Strengths

1. The current systematic review will assess retrospectively the sustainability of differentiated service delivery (DSD) models for HIV treatment in terms of their impact.
2. We aim to equip policy makers with information necessary for making rational choices to prevent wastage of limited resources by providing an evaluation of the sustainability of DSD interventions.

Limitations

3. The comparability of results could be a challenge because a wide range of interventions have been reported and consequently a wide range of outcomes are expected to be included.
4. We will review interventions conducted in SSA and articles published in English leading to a possible bias by excluding articles in other languages.

For peer review only

For peer review only - http://bmjopen.bmj.com/site/about/guidelines.xhtml

INTRODUCTION

In 2015, the World Health Organisation (WHO) recommended treatment initiation for people living with HIV (PLHIV), a 'universal test and treat' strategy. As a result, the number of PLHIV needing antiretroviral treatment (ART) in sub-Saharan Africa (SSA) more than doubled from 12 million to over 25 million [1,2]. This increase in demand adds strain on already weak health systems. It made obvious the insufficiency of traditional clinic-based care and called for innovative service delivery models. Differentiated service delivery (DSD) prioritises client preferences while also aiming to increase efficiency [3–8]. DSD has evolved over time and encompasses a wide range of concepts in program implementation especially in low- and middle-income countries (LMIC) such as task shifting, decentralization, and community-based ART. Ultimately, the goal of DSD is to increase access to and retention in care by reducing demand- and supply-side barriers of clinic-based models of service delivery, especially for rural populations [9–12].

Differentiated care became necessary as more clients were initiated on lifesaving antiretrovirals and ART clinics were becoming crowded. This largely influenced the public health approach involving decentralization recommended by WHO as far back as 2006 [13,14]. Since then, attempts have been made to streamline services by categorizing clients in terms of their clinical profile, such as being 'stable'. Currently, WHO defines a stable client as: "being on ART for at least one year, no current illnesses or pregnancy, a good understanding of lifelong adherence and evidence of treatment success (two consecutive viral load measurements below 1,000 copies/mL)"[1].

Four DSD models were recently described which focus on stable ART clients [15,16]. They include (a) healthcare worker (HCW) groups, (b) facility-based individual models, (c) client managed groups and (d) out-of-facility individual models. Each model is defined by four dimensions, namely 'who' provides care, 'what' care is provided, 'when' do the activities happen, and 'where' is care provided. Pre-packaged ART refill, symptom screening, health talks, clinical consultations or referral if unwell defines 'what' care is provided across models. 'When' care is provided, as a major benefit of DSD, is usually less frequent than the routine monthly visits for clients categorized as stable. Though WHO recommends three to six month intervals, anything between two and 12 month is possible depending on the context. But these models are characterized by the other two dimensions i.e. 'who' provides care (the cadre of healthcare personnel involved) and 'where' care is provided (location). Interventions may be conducted by HCWs within facilities e.g. adherence clubs (ACs), fast track ART refill (FTA), six-monthly appointments (SMA), multi-month scripting (MMS) or by community-based HCWs e.g. community drug distribution points (CDDP). Interventions can also be conducted by non-HCWs within facilities e.g. ACs or by community based non-HCWs e.g. Community ART refill groups (CARG), Community ART groups (CAG), Mobile outreach (OR) and AC (see Table 1 - [17]).

Table 1: Characterization of DSD models

For peer review only - http://bmjopen.bmj.com/site/about/guidelines.xhtml

Location (Where)

		Within facilities	Community based
Personnel (Who)	HCW-managed	Fast track ART refill (FTR)<br>Six monthly appointment (SMA)<br>Multi-month scripting (MMS)<br>Adherence clubs (AC)<br>(Model 1)	Adherence clubs (AC)<br>Community drug distribution points (CDDP)<br>(Model 2)
	Non-HCW managed	Adherence clubs (AC)<br>(Model 3)	Community ART groups (CAG)<br>Community ART refill groups (CARG)<br>Adherence clubs (AC)<br>Mobile outreach (OR)<br>(Model 4)

The potential for achieving a lasting impact and sustainability is the push behind DSD promotion. Sustainability in health care is a broad concept which was only recently defined as "program, clinical intervention, and/or implementation strategies which continue to be delivered after a defined period of time (i.e. after expiration of initial external funding) including the maintenance of program or individual behaviour change (i.e., clinician, client) which may evolve or adapt while continuing to produce benefits for individuals/systems" [18]. Several conceptual frameworks have been described articulating the constructs mentioned in this definition [19–25]. These frameworks consolidate and elaborate on sustainability as an important concept embedded within the continuum of implementation science [26–28]. Most of them view sustainability as a process to be prospectively explored during implementation rather than an outcome to be retrospectively evaluated. Lennox et al [29], in a recent review, developed a comprehensive framework of 40 individual sustainability constructs categorized into six broad domains, namely (1) intervention design and delivery, (2) intervention processes, (3) external environment, (4) organizational setting, (5) resources and (6) people involved. The Lennox framework is the basis used in this review.

Why is it important to do this review?

The long-term sustainability of HIV program interventions is essential as lifelong ART remains a necessity for PLHIV. Therefore, sustainability underlies strategies endorsed by HIV programs e.g. task shifting, decentralization of care , differentiation of care [15,30–35]. The increasing international call for universal health coverage is similarly motivated (in addition to other considerations such as equity) and will likely benefit not just HIV program but entire health systems [36,37]. While many DSD interventions have been implemented within HIV program with good outcomes, knowledge about their sustainability has been limited [38–40]. Furthermore, it is not clear how these interventions can be properly integrated within the existing health system to ensure they last.

Research on the sustainability of health interventions has been hampered by a lack of consensus. For example, several definitions have existed, some referring to the continuation of an intervention as a

For peer review only - http://bmjopen.bmj.com/site/about/guidelines.xhtml

whole or in part [41] and others to the continuation of benefits of an intervention [42]. Similarly, several sustainability frameworks exist, making comparability across studies difficult. Common grounds are however beginning to emerge. The framework by Shediac-Rizkallah [43], provided the basic constructs on which other sustainability studies continue to build [44–46]. The recent syntheses of a comprehensive definition and framework, both anchored in this framework, provided useful tools which we have adopted to gain insight into the sustainability of DSD models [18,29].

In resource constrained settings, the promising client-related outcomes and the reduced demand on resources it places on health systems without undermining effectiveness are thought to make DSD feasible to implement. HIV programs will therefore require evidence to inform which DSD options to explore. Our review aims to fill this gap by evaluating the sustainability of available DSD interventions.

While DSD interventions may not be as onerous as standard clinical care, they pose some demands to the health system. As countries in SSA strive to increase their domestic share of funding for their HIV programs, they must be aware of the aspects influencing sustainability of the DSD options considered. This review will provide information necessary to inform these discussions. Our review also aims to provide the impetus for further enquiry into service delivery models and socio-economic issues necessary to ensure continual client empowerment while supporting minimally resourced health systems.

METHODS AND ANALYSIS

Protocol registration

This review protocol has been registered in the PROSPERO database (CRD42019120891). The protocol adheres to the Preferred Reporting Items for Systematic Reviews and Meta-Analyses-Protocols (PRISMA-P) guidelines [47].

Search strategy

We will search PubMed and EMBASE to identify eligible articles using a comprehensive search strategy (see Supplementary File I). Additionally, the reference lists of included articles and the reference lists of previously published reviews will be reviewed for other relevant articles. The review period of interest will be between 2000 and 2019 to capture all articles published since the widespread scale-up of ART services across SSA. Only English language articles will be included. We will use Rayyan QCRI, the free systematic reviews web application [48] to store, organise and manage eligible references whose titles and abstracts will be screened. A follow-up search will be conducted prior to the completion of the review which is planned nine months after commencement.

Eligibility criteria

The inclusion and exclusion criteria of articles are as listed – see Box 1. Appropriate definitions to guide the selection of articles are also provided – see Box 2.

For peer review only - http://bmjopen.bmj.com/site/about/guidelines.xhtml

Box 1: Eligibility criteria

Inclusions –

- Observational, experimental or quasi-experimental studies.
- Studies involving stable adult ART clients accessing HIV care in SSA.
- Studies describing or assessing HIV services delivered through models other than standard clinic-based care
- Studies which compare the performance of these other service delivery models with standard clinic-based HIV service delivery accessed by other clients. Though, lack of this comparison is not an exclusion criterion.

Exclusions –

- Reviews, editorials, protocol studies and clinical guidelines
- Studies describing or assessing interventions focussed on special population groups e.g. adolescents, children, pregnant women, men who have sex with men, commercial sex workers etc
- Studies utilising data retrospectively collected in electronic databases with little description of the actual intervention

Box 2: Definitions

Stable ART clients

A stable ART client has been changed over time, therefore, there will be no restriction in terms of definition. We will rely on the studies' definitions and will provide a summary of definitions employed.

Adult clients

The definition of adult clients varies across studies. We will define adult as ≥18 years but will include studies if the age-range of participants is not < 15 years

Selection of studies

Two reviewers (NEO and UL) will independently screen the titles and abstracts of articles identified in the searches to identify potential articles. The reviewers will then conduct full-text reading of these potential articles to determine eligibility. GBG and SMH will be engaged in discussions to make a final determination on eligibility whenever the two reviewers are unable to reach a consensus. Reasons for exclusions will be reported.

Data extraction

We have developed a data extraction form. NEO and UL will pilot the data extraction form on a subset of five articles to assess its functionality. After incorporating feedback obtained from the review team (NEO, UL, GBG, SMH) a final form will then be used to extract relevant data independently by the two reviewers.

Data to be extracted will include (a) details of each intervention including site, setting, service delivery model, funding, providers, period, theoretical underpinning; (b) study population, participant type and numbers; (c) sustainability constructs as described in Table 2; (d) challenges or un-intended consequences faced during implementation, comments, clients and Staff perspectives (see Supplementary File II).

We speculate that information regarding some constructs in the framework may be under-reported in the included articles. However, by describing the extent to which this under-reporting is happening, we hope to highlight this issue for further research. We will quantify and report the levels of missing information observed during data extraction (estimated by the number of "not described" assigned by reviewers) per construct for included studies.

For peer review only - http://bmjopen.bmj.com/site/about/guidelines.xhtml

Table 2: Sustainability constructs and evidence to be extracted (adapted from [29]) (Primary study outcomes)

Domain	Item	Sustainability construct	Outcome	Evaluation Question
**The Intervention Design and Delivery**	1	Demonstrating effectiveness	Patient-related outcomes	Does the paper report any numeric or subjective patient-centred outcomes to show effectiveness e.g. retention-in-care, viral suppression, loss-to-follow-up, patient satisfaction
	2	Evidence base for the intervention	Evidence base	Is there evidence that the intervention provides the expected benefits as planned i.e. that the DSD improves outcomes
	3	Expertise	Expertise	Is there evidence of adequate expert knowledge and experience to carry out the DSD especially by supporting organisation
	4	Quality Improvement (QI) methods	QI methods	Is there evidence that QI methods i.e. using data to identify gaps which are continually improved, starting with a pilot and then spreading etc. are used to support intervention success and sustainability
	5	Monitoring progress	Monitoring progress	Is there a standardized and systematic method to gather and report data during DSD intervention
	6	Intervention duration	Duration	Is there evidence that the intervention will last beyond initial funding
	7	Intervention type	Project Design	What type of intervention is it e.g. prevention, treatment, palliative, supportive care
	8	The problem	Problem awareness	Is there general awareness of a problem among stakeholders that requires the DSD intervention to address
	9	Training & capacity building	Capacity building	Is there evidence of any orientation, training, on-going mentoring for staff delivering the DSD intervention
**The External Environment**	10	Awareness and raising the profile	Community awareness	Is there evidence of the larger community being aware of the DSD intervention and promoting its benefit
	11	Socioeconomic and political considerations	Political support	Is there evidence that the intervention has political support? e.g. government engagement, guidelines revision to include DSD requirement etc.
	12	Spread to other organizations	Spread	Is there evidence that the intervention or underlying concepts spread within participating organisation or to other locations
	13	Urgency	Urgency	Is there evidence of an urgency to maintain the intervention based on its relevance
**Intervention processes**	14	Accountability of roles and responsibilities	Roles & responsibilities	Is there evidence that roles & responsibilities of staff involved in the DSD are spread out and clearly defined
	15	Belief in the intervention	Belief in intervention	Is there evidence that staff think the DSD intervention is a better way to do things

For peer review only - http://bmjopen.bmj.com/site/about/guidelines.xhtml

	16	Complexity	Complexity	Is there evidence that it is not difficult for staff to understand and conduct the intervention
	17	Defining aims and shared Vision	Shared goal	Is there evidence of a shared aim and vision established with all stakeholders before commencing the intervention
	18	Incentives	Motivation	Is there evidence that rewards, or benefits derived from the DSD intervention are considered enough motivation that drive stakeholders to engage and continue delivering intervention over time
	19	Job requirements	Job requirements	Is there evidence of revision of job requirement for key staff incorporating the DSD intervention tasks as part of key job descriptions
	20	Workload	Workload	Is there evidence that any additional workload introduced by the DSD intervention is manageable and requiring no special effort to staff involved
**Resources**	21	General resources	General resources	Is there evidence that resources needed to manage and maintain the DSD intervention are available
	22	Funding	Funding	Is there evidence that adequate funds are available to implement, and strategic funds planned to sustain intervention i.e. DSD will be embedded and sustained
	23	Infrastructure	Infrastructure	Is there evidence that resources required to support the DSD intervention e.g. office space, materials, and supplies are available
	24	Staff	Staff	Is there evidence of enough staff in place to conduct and sustain DSD intervention
	25	Time	Time	Is there evidence that adequate time was dedicated for DSD intervention in the routine daily schedule of the facility
**Organizational setting**	26	Integration with existing programs and policies	Integration	Is there evidence that DSD intervention was embedded within the existing organizational structure, programs and policies
	27	Intervention adaptation and receptivity	Adaptation	Is there evidence that the DSD intervention is flexible to respond, change, adapt and fit with local context requirement
	28	Opposition	Opposition	Is there evidence of any resistance due to other competing interests from stakeholders reported
	29	Organizational readiness and capacity	Readiness	Is there evidence that health facilities have adequate capacity and readiness to undertake the DSD intervention i.e. in terms of materials and manpower
	30	Organizational values and culture	Values & culture	Is there evidence that the values of the DSD intervention align with health system values, prevailing beliefs and culture and priorities
	31	Support available	Management support	Is there evidence of facility management support for the delivery and maintenance of the DSD intervention

For peer review only - http://bmjopen.bmj.com/site/about/guidelines.xhtml

**The people involved**	32	Leadership and champions	Champions	Is there evidence of any influential person or group who advocates and supports the DSD intervention
	33	Ownership	Ownership	Is there evidence that stakeholders take ownership to support, embed and sustain the DSD intervention
	34	Power	Power	Is there evidence that stakeholders can use their power to make decisions, advocate and support the DSD intervention
	35	Relationships, collaboration, networks	Collaboration	Is there evidence of any collaborations, partnerships and support networks to promote and sustain the DSD intervention
	36	Satisfaction	Satisfaction	Is there evidence of benefits and rewards enjoyed by stakeholders and staff for participation in DSD intervention reported
	37	Stakeholder participation	Stakeholder participation	Is there evidence that key stakeholders (those affected by the intervention) are engaged and participate in DSD intervention
	38	Community participation	Community participation	Is there evidence of the participation of community members in directing and shaping DSD intervention goals and approaches to reflect their values and needs
	39	Patient involvement	Patient involvement	Is there evidence of the involvement of patients in DSD intervention processes to understand patient's perspectives, values and needs
	40	Staff involvement	Staff involvement	Is there evidence of the involvement of staff in the planning, design, delivery of the DSD intervention

For peer review only - http://bmjopen.bmj.com/site/about/guidelines.xhtml

Data Analysis

Primary outcome measures will include the scores and rankings on sustainability constructs and domains as defined by Lennox et al [29] and presented in Table 2. Since the review aims to extract and appraise evidence of sustainability constructs retrospectively, we simplified a previously described tool to assess sustainability in on-going projects [49]. The two reviewers will assign a score of 3, 2 or 1 independently based on their assessment of whether there was enough, some or no evidence about each construct (see Supplementary file II). Additionally, “not described” (ND) will be assigned if the reviewers are not able to make an assessment with information provided. Attempts will be made to contact study authors directly for any additional data considered necessary. Secondary outcome measures will include (a) trends observed in sustainability scores as a result of variations in the criteria employed to define stable clients, (b) if appropriate, quantitative intervention outcome measures such as retention in care, viral suppression, loss-to-follow-up, client or provider costs reported across similar interventions, and (c) qualitative outcomes such as perspectives of clients on issues such as satisfaction with intervention or HCW, access to ART, peer support and perspectives of HCWs on workload, or care for sick clients among others. Qualitative outcomes will be extracted as text.

Qualitative and quantitative analyses will be conducted. The basic characteristics, key findings including strengths and challenges of DSD interventions will be summarised. For qualitative analysis, we will conduct narrative synthesis [50] to identify commonly occurred themes. For quantitative analysis, we will employ descriptive statistics to determine the sustainability performance of DSD interventions. Performance will be measured in terms of overall construct scores and percentages and median domain scores. This will then be used to rank constructs, domains, interventions and models for sustainability with higher scores ranked as better sustainability.

Quality of evidence

Two reviewers, NEO and UL, will assess risk of bias in included studies using the Downs and Black checklist [51]. The following domains will be assessed: quality of reporting, external validity and internal validity, confounding (selection bias) and power. For qualitative studies, we will assess risk of bias using the Joanna Briggs Institute (JBI) checklist [52]. Disagreements between reviewers will be resolved as for study selection. The overall quality of included studies will be evaluated, if possible, using the Grading of Recommendations Assessment, Development and Evaluation (GRADE) framework for quality of evidence [53].

ETHICS AND DISSEMINATION

No ethical approval is required for the conduct of this systematic review since all data used are available in the public space. Results and findings of the review will be published in a peer-reviewed journal and presented at appropriate conferences and meetings.

For peer review only - http://bmjopen.bmj.com/site/about/guidelines.xhtml

AUTHORS' CONTRIBUTIONS

NEO, GBG and SH conceived the review design and in refining and registering the review protocol. NEO, GBG, SH and UL contributed to the search strategy and to developing the data extraction form. NEO drafted the initial manuscript. NEO, GBG, SH, DN and TRW provided content expertise. All authors contributed to revisions to obtain the final manuscript. All authors read and approved the final manuscript.

PATIENT AND PUBLIC INVOLVEMENT STATEMENT

We will be reviewing and assessing already conducted interventions retrospectively and this will permit little or no involvement of patients and the public.

FUNDING STATEMENT

This work was supported by Erasmus Mundus Joint Doctorate Fellowship, Framework Partnership Agreement 2013-0039, Specific Grant Agreement 2014-0681.

COMPETING INTERESTS' STATEMENT

No competing interests declared

For peer review only

For peer review only - http://bmjopen.bmj.com/site/about/guidelines.xhtml

REFERENCES

1 World Health Organization. Consolidated guidelines on the use of antiretroviral drugs for treating and preventing HIV infection: recommendations for a public health approach. *World Heal Organ* 2016;:155 p. doi:10.1016/j.jped.2014.04.007

2 Pustil R. Global AIDS Update. *Aids* 2016;**17 Suppl 4**:S3-11. doi:10.1073/pnas.86.15.5781

3 Bango F, Ashmore J, Wilkinson L, *et al.* Adherence clubs for long-term provision of antiretroviral therapy: cost-effectiveness and access analysis from Khayelitsha, South Africa. *Trop Med Int Heal* 2016;**21**:1115–23. doi:10.1111/tmi.12736

4 Long L, Brennan A, Fox MP, *et al.* Treatment Outcomes and Cost-Effectiveness of Shifting Management of Stable ART Patients to Nurses in South Africa: An Observational Cohort. *PLoS Med* 2011;**8**:e1001055. doi:10.1371/journal.pmed.1001055

5 Babigumira JB, Castelnuovo B, Stergachis A, *et al.* Cost effectiveness of a Pharmacy-only Refill Program in a large Urban HIV/AIDS clinic in Uganda. *PLoS One* 2011;**6**:e18193. doi:10.1371/journal.pone.0018193

6 Grimsrud A, Bygrave H, Doherty M, *et al.* Reimagining HIV service delivery: The role of differentiated care from prevention to suppression: The. J. Int. AIDS Soc. 2016;**19**. doi:10.7448/IAS.19.1.21484

7 Okoboi S, Ding E, Persuad S, *et al.* Community-based ART distribution system can effectively facilitate long-term program retention and low-rates of death and virologic failure in rural Uganda. *AIDS Res Ther* 2015;**12**. doi:10.1186/s12981-015-0077-4

8 Tsondai PR, Wilkinson LS, Grimsrud A, *et al.* High rates of retention and viral suppression in the scale-up of antiretroviral therapy adherence clubs in Cape Town, South Africa. *J Int AIDS Soc* 2017;**20**. doi:10.7448/IAS.20.5.21649

9 Bedelu M, Ford N, Hilderbrand K, *et al.* Implementing Antiretroviral Therapy in Rural Communities: The Lusikisiki Model of Decentralized HIV/AIDS Care. *J Infect Dis* 2007;**196**:S464–8. doi:10.1086/521114

10 Fredlund VG, Nash J. How Far Should They Walk? Increasing Antiretroviral Therapy Access in a Rural Community in Northern KwaZulu-Natal, South Africa. *J Infect Dis* 2007;**196**:S469–73. doi:10.1086/521115

11 Mutevedzi PC, Lessells RJ, Heller T, *et al.* Scale-up of a decentralized HIV treatment programme in rural KwaZulu-Natal, South Africa: does rapid expansion affect patient outcomes? *Bull World Health Organ* 2010;**88**:593–600. doi:10.2471/BLT.09.069419

12 Davis N, Kanagat N, Sharer M, *et al.* Review of differentiated approaches to antiretroviral therapy distribution. AIDS Care - Psychol. Socio-Medical Asp. AIDS/HIV. 2018;:1–7. doi:10.1080/09540121.2018.1441970

13 WHO Guidelines. *ANTIRETROVIRAL THERAPY FOR HIV INFECTION IN ADULTS AND ADOLESCENTS: Recommendations for a public health approach HIV/AIDS Programme IMPORTANT: ADDENDUM TO 2006 WHO GUIDELINES ON ANTIRETROVIRAL THERAPY FOR HIV INFECTION IN ADULTS AND ADOLESCENTS*. WHO 2006.

14 Gilks CF, Crowley S, Ekpini R, *et al.* The WHO public-health approach to antiretroviral treatment against HIV in resource-limited settings. *Lancet* 2006;**368**:505–10. doi:10.1016/S0140-6736(06)69158-7

15 IAS. Differentiated Care for Hiv: a Decision Framework for Antiretroviral Therapy Delivery. *Differ Care.org (International AIDS Soc* 2016;:1–56.

16 WHO. Key considerations for differentiated antiretroviral therapy delivery for specific populations: children, adolescents, pregnant and breastfeeding women and key populations. 2017.

17 International AIDS Society I. Differentiated Service Delivery. IAS.

For peer review only - http://bmjopen.bmj.com/site/about/guidelines.xhtml

2019.http://www.differentiatedcare.org/ (accessed 3 Jun 2019).
18 Moore JE, Mascarenhas A, Bain J, *et al.* Developing a comprehensive definition of sustainability. *Implement Sci* 2017;**12**:110. doi:10.1186/s13012-017-0637-1
19 Whelan J, Love P, Pettman T, *et al.* Cochrane Update Predicting sustainability of intervention effects in public health evidence: identifying key elements to provide guidance. doi:10.1093/pubmed/fdu027
20 Schell SF, Luke DA, Schooley MW, *et al.* Public health program capacity for sustainability: A new framework. *Implement Sci* 2013;**8**:1. doi:10.1186/1748-5908-8-15
21 Gruen RL, Elliott JH, Nolan ML, *et al.* Sustainability science: an integrated approach for health-programme planning. *Lancet* 2008;**372**:1579–89. doi:10.1016/S0140-6736(08)61659-1
22 Hodge LM, Turner KMT. Sustained Implementation of Evidence-based Programs in Disadvantaged Communities: A Conceptual Framework of Supporting Factors. *Am J Community Psychol* 2016;**58**:192–210. doi:10.1002/ajcp.12082
23 Mancini JA, Marek LI. Sustaining Community-Based Programs for Families: Conceptualization and Measurement*. *Fam Relat* 2004;**53**:339–47. doi:10.1111/j.0197-6664.2004.00040.x
24 Sarriot EG, Winch PJ, Ryan LJ, *et al.* A methodological approach and framework for sustainability assessment in NGO-implemented primary health care programs. *Int J Health Plann Manage* 2004;**19**:23–41. doi:10.1002/hpm.744
25 Luke DA, Calhoun A, Robichaux CB, *et al.* The Program Sustainability Assessment Tool: a new instrument for public health programs. *Prev Chronic Dis* 2014;**11**:130184. doi:10.5888/pcd11.130184
26 Moullin JC, Sabater-Hernández D, Fernandez-Llimos F, *et al.* A systematic review of implementation frameworks of innovations in healthcare and resulting generic implementation framework. *Heal Res Policy Syst* 2015;**13**. doi:10.1186/s12961-015-0005-z
27 Feldstein AC, Glasgow RE. A practical, robust implementation and sustainability model (PRISM) for integrating research findings into practice. *Jt Comm J Qual Patient Saf* 2008;**34**:228–43. doi:10.1016/S1553-7250(08)34030-6
28 Theobald S, Brandes N, Gyapong M, *et al.* Implementation research: new imperatives and opportunities in global health. *Lancet* 2018;**392**:2214–28. doi:10.1016/S0140-6736(18)32205-0
29 Lennox L, Maher L, Reed J. Navigating the sustainability landscape: A systematic review of sustainability approaches in healthcare. Implement. Sci. 2018;**13**:27. doi:10.1186/s13012-017-0707-4
30 Callaghan M, Ford N, Schneider H. A systematic review of task- shifting for HIV treatment and care in Africa. Hum. Resour. Health. 2010;**8**:8. doi:10.1186/1478-4491-8-8
31 Philips M, Zachariah R, Venis S. Task shifting for antiretroviral treatment delivery in sub-Saharan Africa: not a panacea. *Lancet* 2008;**371**:682–4. doi:https://dx.doi.org/10.1016/S0140-6736(08)60307-4
32 Kredo T, McCaul M, Volmink J. Task-shifting from doctors to nondoctors for initiation and maintenance of antiretroviral therapy. South African Med. J. 2015;**105**:626–7. doi:10.7196/SAMJnew.8271
33 Pasipamire L, Nesbitt RC, Ndlovu S, *et al.* Retention on ART and predictors of disengagement from care in several alternative community-centred ART refill models in rural Swaziland. Published Online First: 2018. doi:10.1002/jia2.25183/full
34 Adjetey V, Obiri-Yeboah D, Dornoo B. Differentiated service delivery: A qualitative study of people living with HIV and accessing care in a tertiary facility in Ghana. *BMC Health Serv Res* 2019;**19**. doi:10.1186/s12913-019-3878-7
35 Prust ML, Banda CK, Nyirenda R, *et al.* Multi-month prescriptions, fast-track refills, and community ART groups: results from a process evaluation in Malawi on using differentiated

For peer review only - http://bmjopen.bmj.com/site/about/guidelines.xhtml

models of care to achieve national HIV treatment goals. *J Int AIDS Soc* 2017;**20**:21650. doi:10.7448/IAS.20.5.21650
36 World Health Organisation (WHO). Hiv, Universal Health Coverage and the Post-2015 Development Agenda. 2015.
37 The Global Fund. Building Resilient and Sustainable Systems for Health. 2018.
38 Castelli F, Pietra V, Diallo I, *et al.* Antiretroviral (ARV) Therapy in Resource Poor Countries: What do we Need in Real Life? *Open AIDS J* 2010;**4**:28–32. doi:10.2174/1874613601004020028
39 Azeredo TB, Oliveira MA, Santos-Pinto CDB, *et al.* Sustainability of ARV provision in developing countries: challenging a framework based on program history. *Cien Saude Colet* 2017;**22**:2581–94. doi:10.1590/1413-81232017228.29472016
40 Abebe B. Sustainability of HIV/AIDS Care & Support Programmes Acknowledgement-Igratefully acknowledge Prof. Yemane Berhane, program and theory review course. *Type Double Blind Peer Rev Int Res J Publ Glob Journals Inc* 2012;**12**.
41 Ament SMC, De Groot JJA, Maessen JMC, *et al.* Sustainability of professionals' adherence to clinical practice guidelines in medical care: A systematic review. BMJ Open. 2015;**5**:e008073. doi:10.1136/bmjopen-2015-008073
42 Wiltsey Stirman S, Kimberly J, Cook N, *et al.* The sustainability of new programs and innovations: a review of the empirical literature and recommendations for future research. *Implement Sci* 2012;**7**:17. doi:10.1186/1748-5908-7-17
43 Shediac-Rizkallah MC, Bone LR. Planning for the sustainability of community-based health programs: conceptual frameworks and future directions for research, practice and policy. 1998.
44 Chambers DA, Glasgow RE, Stange KC. The dynamic sustainability framework: addressing the paradox of sustainment amid ongoing change. *Implement Sci* 2013;**8**:1. doi:10.1186/1748-5908-8-117
45 Scheirer MA. Is Sustainability Possible? A Review and Commentary on Empirical Studies of Program Sustainability. *Am J Eval* 2005;**26**:320–47. doi:10.1177/1098214005278752
46 Iwelunmor J, Blackstone S, Veira D, *et al.* Toward the sustainability of health interventions implemented in sub-Saharan Africa: a systematic review and conceptual framework. *Implement Sci* 2016;**11**. doi:10.1186/s13012-016-0392-8
47 Moher D, Shamseer L, Clarke M, *et al.* Preferred reporting items for systematic review and meta-analysis protocols (PRISMA-P) 2015 statement. Published Online First: 2015. doi:10.1186/2046-4053-4-1
48 Ouzzani M, Hammady H, Fedorowicz Z, *et al.* Rayyan-a web and mobile app for systematic reviews. *Syst Rev* 2016;**5**:1–10. doi:10.1186/s13643-016-0384-4
49 Calhoun A, Mainor A, Moreland-Russell S, *et al.* Using the Program Sustainability Assessment Tool to assess and plan for sustainability. *Prev Chronic Dis* 2014;**11**:130185. doi:10.5888/pcd11.130185
50 Popay J, Roberts H, Sowden A, *et al.* Guidance on the Conduct of Narrative Synthesis in Systematic Reviews. *A Prod from ESRC Methods Program* 2006;:211–9. doi:10.1111/j.1523-536x.1995tb00261.x
51 Downs SH, Black N. The feasibility of creating a checklist for the assessment of the methodological quality both of randomised and non-randomised studies of health care interventions. *J Epidemiol Community Heal* 1998;**52**:377–84.
52 Lockwood C, Munn Z, Porritt K. Qualitative research synthesis. *Int J Evid Based Healthc* 2015;**13**:179–87. doi:10.1097/XEB.0000000000000062
53 Guyatt G, Oxman AD, Akl EA, *et al.* GRADE guidelines: 1. Introduction - GRADE evidence profiles and summary of findings tables. *J Clin Epidemiol* 2011;**64**:383–94. doi:10.1016/j.jclinepi.2010.04.026

For peer review only

For peer review only - http://bmjopen.bmj.com/site/about/guidelines.xhtml

Supplementary File I: Search strategy (PubMed & EMBASE)

#1 Pubmed Search ALL <2000 to November 21, 2019>

Search history sorted by search number ascending

#	Searches
**1**	(patient-centred care or patient-centered care or community supported models or adherence clubs or teen* club or task shifting or ("community ART" adj2 (distribution or delivery or refill)) or community client lead ART-delivery or facility fast track or quick pick-up or (p?ediatric expedited adj2 ART)).mp. or ((differentiated or decentrali?ed or community) adj2 (care or intervention)).ti,ab,kf. or (adherence club or differentiated model* or down-referr* or out-of-clinic).ti,ab,kf.
**2**	exp HIV/ or exp HIV INFECTIONS/ or exp HIV Seropositivity/ or hiv.ti,ab,kf. or (antiretroviral treatment or ART).ti,ab,kf.
**3**	1 and 2

#2 Embase search <2000 to November 21, 2019>

Search history sorted by search number ascending

#	Searches
**1**	(patient-centred care or patient-centered care or community supported models or adherence clubs or teen* club or task shifting or ("community ART" adj2 (distribution or delivery or refill)) or community client lead ART-delivery or facility fast track or quick pick-up or (p?ediatric expedited adj2 ART)).mp. or ((differentiated or decentrali?ed or community) adj2 (care or intervention)).ti,ab,kw. or (adherence club or differentiated model* or down-referr* or out-of-clinic).ti,ab,kw.
**2**	exp Human immunodeficiency virus/
**3**	exp Human immunodeficiency virus infection/
**4**	(hiv or (antiretroviral treatment or ART)).ti,ab,kw.
**5**	2 or 3 or 4
**6**	1 and 5
**7**	limit 6 to (conference abstracts or embase)

Supplementary File II: Data extraction tool

Publication detail		Setting		Intervention detail																	Study detail		Quantitative Outcomes - any numeric patient-centered outcomes to show effectiveness (*Insert reported outcome values/units/definition only*)																							
Author, Year, Journal	Title of publication	Town, Country e.g. Tete, Mozambique	Setting i.e. Rural or Urban	HIV project/Study site e.g. MSF project	Project ownership i.e. Government (MOH)or NGO (MSF)	Year of commencement of Intervention	Who is the Target population/ Intervention group	Comparison group/contemporary or historical	Definition of "Stable" patient	Number of participants (stable)	Number of participants (comparison)	Model e.g. Decentralized care, DCM (if so which? Task shifting	Brief description of intervention	Level of implementation (number of sites)	Study aim	Study variables definitions	Primary care provider in intervention	Primary care provider in standard of care (SOC)	Provider in intervention funded by	Provider in SOC funded by	Study design	Any Theoretical underpinning for the study	Outcomes - VL (%) SOC	Outcomes - VL (%) DCM intervention	Outcome - VL (rate) SOC	Outcome - VL (rate) DCM intervention	*Comments about units or subgroup analysis*	Outcome - Retention (%)SOC	Outcome - Retention (%)DCM intervention	*Comments about units or any subgroup analysis*	Outcome - LTFU (%)SOC	Outcome - LTFU (%)DCM intervention	Outcome - LTFU (rate) SOC	Outcome - LTFU (rate)	*Comments about units or any subgroup analysis*	Outcome - Mortality (%) SOC	Outcome - Mortality (%) DCM intervention)	Outcome - Mortality (rate)SOC	Outcome - Mortality (rate)DCM intervention	*Comments about units or any subgroup analysis*	Outcome - Provider Cost/visit ($)SOC	Outcome - Provider Cost/visit ($)DCM intervention)	Outcome - Patient. Cost/visit ($)SOC	Outcome - Patient. Cost/visit ($)DCM intervention)	*Comments about units or any subgroup analysis*

Intervention Design and Delivery								External environment				Intervention Process					
**Evidence base** - Is there evidence that the intervention provides the expected benefits as planned i.e. that the DSD improves outcomes	**Expertise** - Is there evidence of adequate expert knowledge and experience to carry out the DSD especially by supporting organisation	**QI methods** - Is there evidence that QI methods i.e. using data to identify gaps which are continually improved, starting with a pilot and then spreading etc. are used to support intervention success and sustainability	**Monitoring progress** - Is there a standardized and systematic method to gather and report data during DSD intervention	**Project duration** - Is there evidence that the intervention will last beyond initial funding	**Project type** - What type of intervention is it e.g. prevention, treatment, palliative, supportive care	**Problem** - Is there general awareness of a problem among stakeholders that requires the DSD intervention to address	**Capacity building** - Is there evidence of any orientation, training, on-going mentoring for staff delivering the DSD intervention	**Awareness** - Is there evidence of the larger community being aware of the DSD intervention and promoting its benefit	**Political support** - Is there evidence that the intervention has political support? e.g. government engagement, guidelines revision to include DSD requirement etc.	**Spread** - Is there evidence that the intervention or underlying concepts spread within participating organisation or to other locations	**Urgency** - Is there evidence of an urgency to maintain the DSD intervention based on its relevance	**Roles & responsibilities** - Is there evidence that roles & responsibilities of staff involved in the DSD are spread out and clearly defined	**Belief in initiative** - Is there evidence that staff think the DSD intervention is a better way to do things	**Complexity** - Is there evidence that it is not difficult for staff to understand and conduct the DSD intervention	**Shared goal** - Is there evidence of a shared aim and vision established with all stakeholders before commencing the DSD intervention	**Incentives** - Is there evidence that rewards or benefits derived from the DSD intervention are considered enough motivation that drive stakeholders to engage and continue delivering intervention over time	**Job requirement /descriptions** - Is there evidence of revision of job requirement for key staff incorporating the DSD intervention tasks as part of key job descriptions
3 = There is sufficient evidence that the intervention provides expected benefits to stakeholders	3 = There is sufficient evidence that the supporting organization has adequate expert knowledge and experience to conduct intervention	3 = There is sufficient evidence of the use of QI methods to support the conduct of intervention	3 = There is sufficient evidence of monitoring the intervention using standardised system to gather and report data over time	3 = There is sufficient evidence the intervention will last for a long time beyond the initial project and funding	3 = The type and design of the project is clear (e.g. preventive, treatment, palliative care, supportive care etc.)	3 = The intervention is addressing a problem that is recognised and accepted as a real concern by all stakeholders i.e. community, staff, patients, supporting organisation, facility, government	3 = There is sufficient evidence of orientation/ training/ mentoring to new staff and on-going training to all staff to be able to deliver intervention successfully	3 = There is sufficient evidence of effort to ensure that stakeholders e.g. the community are aware of the benefits of the intervention through media marketing e.g. patient pressure groups, community leaders	3 = There is sufficient evidence the intervention has the full support of Government (involvement of the MOH, District/State/zonal health unit, institutions, Revision of guideline, inclusion in strategic plans etc.)	3 = There is sufficient evidence that the intervention or beneficial parts of it are spread within a facility or to other facilities in a community or district	3 = There is sufficient evidence of motivation or urgency to maintain the intervention or parts of it based on its perceived potential of supporting a relevant healthcare need.	3 = There is sufficient evidence that roles and responsibilities of all staff involved in the intervention is clear and evenly distributed so no staff is over-burdened	3 = There is sufficient evidence that majority of staff conducting the intervention believe the change is a better way of doing thing and will add value	3 = There is sufficient evidence that it is not hard to understand, conduct and maintain the intervention	3 = There is sufficient evidence of a shared aim and vision for the intervention existing among all major stakeholders including the community, government, partners, patients as well as goal revision when necessary	3 = There is sufficient evidence of perceived benefit from the intervention by all stakeholders including community, supporting organisation, patients, staff and government	3 = There is sufficient evidence of revised job requirement for key staff in facilities which capture the roles and job functions introduced by the intervention e.g. revised job description, SOP, guidelines OR a revision in job requirement was not required
2 = There is some evidence that the intervention provides expected benefits to stakeholders	2 = There is some evidence that the supporting organisation has adequate expert knowledge and experience to conduct intervention	2 = There is some evidence of the use of QI methods to support the conduct of intervention	2 = There is some evidence of monitoring the intervention using standardised system to gather and report data over time	2 = There is some evidence the intervention will last for some time beyond the initial project and funding	2 = The type and design of the project is clear to some extent (e.g. preventive, treatment, palliative care, supportive care etc.)	2 = The intervention is addressing a problem that is recognised and accepted as a concern by some stakeholders i.e. mainly by the facility, patients, staff, supporting organization	2 = There is some evidence of orientation/training/ mentoring to new staff and to all staff to be able to deliver intervention successfully	2 = There is some evidence of effort to ensure that stakeholders e.g. the community are aware of the benefits of the intervention through media marketing e.g. community leaders alone or patients or pressure groups	2 = There is evidence the intervention has some level of support of the Government (involvement of the MOH, District/State/zonal health unit, an institution, etc.)	2 = There is some evidence the intervention spread to a few other sites beyond the intervention facility	2 = There is some evidence of motivation or urgency to maintain the intervention or parts of it based on its perceived potential of supporting a relevant healthcare need.	2 = There is some evidence that roles and responsibilities of staff involved in the intervention is clear and evenly distributed so no staff is over-burdened	2 = There is some evidence that staff conducting the intervention believe the change is a better way of doing thing and will add value	2 = There is evidence of some difficulty in understanding or conducting and maintaining the intervention	2 = There is some evidence of a shared aim and vision for the intervention existing among most stakeholders including the community, a local partners and patients.	2 = There is some evidence of perceived benefit from the intervention by some stakeholders e.g. only supporting organisation or patients or staff	2 = There is some evidence of revised job requirement for key staff in facilities with new roles and job functions introduced by the intervention but no revised job descriptions, SOP
1 = There is little or no evidence that the intervention provides expected benefits to stakeholders	1 = There is little or no evidence the supporting organisation has adequate expert knowledge and experience to conduct intervention	1 = There is little or no evidence of the use of QI methods to support the conduct of intervention to a little or no extent	1 = There is little or no evidence of monitoring the intervention using standardised system to gather and report data over time	1 = There is little or evidence the intervention will last beyond the initial project and funding	1 = The type and design of the project is clear to a little extent (e.g. preventive, treatment, palliative care, supportive care etc.)	1 = The intervention is addressing a problem that is not relly recognised and accepted as a concern by any stakeholders	1 = There is little or no evidence of orientation/training/ mentoring to staff to be able to deliver intervention successfully	1 = Only PLHIV and lay workers in the community participating in the intervention are aware of the intervention	1 = There is evidence the intervention has the support of at least the institution involved in implementation, a local NGO etc.	1 = There is evidence the intervention or beneficial parts of it are spread to at least other parts of a facility	1 = There is little or no evidence of motivation or urgency to maintain the intervention or parts of it based on its perceived potential of supporting a relevant healthcare need.	1 = There is little or no evidence that roles and responsibilities of staff involved in the intervention are clear or evenly distributed	1 = There is little or no evidence that staff conducting the intervention believe the change is a better way of doing things	1 = There is evidence of moderate difficulty in understanding or conducting and maintaining the intervention	1 = There is little or no evidence of a shared aim and vision for the intervention existing among stakeholders. Only the supporting partner developed a goal.	1 = There is little or no evidence of perceived benefit from the intervention by any stakeholder, maybe only the supporting organisation	1 = There is little or no evidence of revised job requirement for the staff invloved with implementing the intervention at the facilities

For peer review only

	Resources				
**Additional workload** - Is there evidence that any additional workload introduced by the DSD intervention is manageable and requiring no special effort to staff involved	**Resources - General** - Is there evidence that resources needed to manage and maintain the DSD intervention are available	**Funding** - Is there evidence that adequate funds are available to implement and strategic funds planned to sustain the DSD intervention	**Infrastructure** - Is there evidence that resources required to support the DSD intervention e.g. office space, materials, and supplies are available	**Staff** - Is there evidence of sufficient staff in place to conduct and sustain the DSD intervention	**Time** - Is there evidence that adequate time was dedicated for the DSD intervention in the routine daily schedule of the facility
3 = There is sufficient evidence that any additional workload introduced by the intervention is manageable and evenly divided among staff without requiring extra effort	3 = There is sufficient evidence that all/most resources required to conduct and maintain the intervention are available and adequate and provided by the government i.e. more government less external donor	3 = There is sufficient evidence that adequate (all/most) funds required to implement and sustain the intervention are available and provided by government	3 = There is sufficient evidence that all/most of the resources required to support the intervention such as buildings, office space, materials and supplies are available and provided by the government	3 = There is sufficient evidence of sufficient number of staff, internal and external (i.e. a team) in place to implement and sustain the intervention employed by the government	3 = There is sufficient evidence that adequate time was dedicated to the intervention activities in the routine daily schedule of the facility
2 = There is evidence that the additional workload introduced by the intervention is manageable to a some extent and evenly divided among staff	2 = There is evidence that some resources required to conduct and maintain the intervention are available and adequate i.e. less government and more external donor	2 = There is evidence that most of the funds required to implement and sustain the intervention are available e.g.less government funds and more external donor funds	2 = There is evidence that the resources required to support the intervention such as buildings, office space, materials and supplies are available i.e. less government support and more external donor support	2 = There is evidence that the number of internal staff in place to implement and sustain the intervention is mostly adequate i.e. the cadre of staff needed is employed by govenrment but paid with external donor funds	2 = There is evidence that time was dedicated to some of the intervention activities in the routine daily schedule of the facility
1 = There is little or no evidence that the additional workload introduced by the intervention is manageable to the staff involved	1 = There is evidence that little or none of the resources required to conduct and maintain the intervention are available and adequate i.e. mostly provided by external donor	1 = There is evidence that little or none of the funds required to implement and sustain the intervention are available e.g.mostly provided by external donor funds	1 = There is evidence that the resources required to support the intervention such as buildings, office space, materials and supplies are available and mostly paid by external funder	1 = There is evidence that the number of internal staff in place to implement and sustain the intervention is somewhat adequate i.e. project staff are mainly employed and paid by extarnal funder	1 = There is evidence that time was dedicated to little or none of the intervention activities in the routine daily schedule of the facility i.e. project activities run within the facility but parallel to routine facility activities

Organizational setting					
**Integration** - Is there evidence that the DSD intervention was embedded within the existing organizational structure, Programme and policies	**Adaptation** - Is there evidence that the DSD intervention is flexible to respond, change, adapt and fit with local context requirement	**Opposition** - Is there evidence of any resistance due to other competing interests from stakeholders reported	**Readiness** - Is there evidence that health facilities have adequate capacity and readiness to undertake the DSD intervention i.e. in terms of materials and manpower	**Organizational Value system and culture** - Is there evidence that the values of the DSD intervention align with health system values, prevailing beliefs and culture and priorities	**Management support** - Is there evidence of facility management support for the delivery and maintenance of the DSD intervention
3 = There is sufficient evidence that the intervention was embedded within the existing organizational structures, programmes and policies of the health system and the facility	3 = There is sufficient evidence that the intervention responds to changes and adapts to fit with local context and requirements	3 = There is sufficient evidence of no resistance from stakeholders to the intervention due to other competing priorities	3 = There is sufficient evidence of adequate capacity and readiness of facilities to conduct the intervention i.e. all materials and staff needed are provided by government	3 = There is sufficient evidence that the health system and facility values, prevailing beliefs and culture and priorities support the sustainability and strategic direction of the intervention e.g. inclusion instragegic plan or guideline	3 = There is sufficient evidence of management total support of the intervention in the form of reminders, staff, technical and education to enhance delivery
2 = There is evidence that the intervention was embedded to some extent within the existing organizational structures, programmes and policies of the facility	2 = There is some evidence that the intervention responds to changes and adapts to fit with local context and requirements	2 = There is evidence of some resistance from stakeholders to the intervention due to other competing priorities	2 = There is some evidence of capacity and readiness of facilities to conduct the intervention i.e. most materials and staff needed are provided by government with support from external funder	2 = There is some evidence that the health system and facility values, prevailing beliefs and culture and priorities support the sustainability and strategic direction of the intervention e.g. some government involvement with little commitment	2 = There is some evidence of management support of the intervention to a large extent in the form of reminders, staff, technical and education to enhance delivery i.e. in principle but it is non-commital
1 = There is evidence that the intervention was embedded to a little extent within the existing organizational structures, programmes and policies of the facility	1 = There is little or no evidence that the intervention responds to changes and adapts to fit with local context and requirements	1 = There is evidence of resistance to a large extent from stakeholders to the intervention	1 = There is evidence that capacity and readiness of facilities to conduct the intervention is limited i.e. aii/most materials and staff needed are provided by external funder	1 = There is little or no evidence that the health system and facility values, prevailing beliefs and culture and priorities support the sustainability and strategic direction of the intervention	1 = There is little or no evidence of management support of the intervention

People Involved								
**Leaders/champions** - Is there evidence of any influential person or group who advocates and supports the DSD intervention	**Ownership** - Is there evidence that stakeholders take ownership to support, embed and sustain the DSD intervention	**Power** - Is there evidence that stakeholders have the ability to use their power to make decisions, advocate and support the DSD intervention	**Collaboration** - Is there evidence of any collaborations, partnerships and support networks to promote and sustain the DSD intervention	**Satisfaction** - Is there evidence of benefits and rewards enjoyed by stakeholders and staff for participation in the DSD intervention reported	**Stakeholders participation** - Is there evidence that key stakeholders (those affected by the intervention) are engaged and participate in the DSD intervention	**Community participation** - Is there evidence of the participation of community members in directing and shaping DSD intervention goals and approaches to reflect their values and needs	**Patient involvement** - Is there evidence of the involvement of patients in DSD intervention processes to understand patient's perspectives, values and needs	**Staff involvement** - Is there evidence of the involvement of staff in the planning, design, delivery of the DSD intervention
3 = There is evidence of an influential person (champion) and group of people (patient pressure group) who have the ability and skills to advocate, communicate and support the intervention e.g. a prominent community leader, a PLHIV group, NGO	3 = There is sufficient evidence that the government, facilities, communities and other stakeholders take ownership and responsibility to support the intervention	3 = There is sufficient evidence of the ability of stakeholders to use their power to make decisions, advocate and support initiative	3 = There is sufficient evidence of partnerships, collaborations and networks to support and sustain the intervention e.g. with government and other local stakeholders, patient groups etc.	3 = There is sufficient evidence of acceptance, enjoyment and reward among stakeholders from participating in intervention e.g. Government, local partners, staff, patients etc.	3 = There is sufficient evidence of the involvement and participation of stakeholders who are affected by the intervention e.g. Government, community, staff, patients	3 = There is sufficient evidence of the participation of community members to direct and shape the intervention to reflect their values, expectations and needs e.g. involving community groups and leaders	3 = There is sufficient evidence of the involvement of patients in the intervention's processes, to understand patient's perspective i.e. in the design and process	3 = There is sufficient evidence of the involvement of staff in the planning, design, delivery and maintenance of the intervention
2 = There is some evidence of an influential person (champion) and group of people (patient pressure group) who have the ability and skills to advocate, communicate and support the intervention e.g. expert patients living openly with their status	2 = There is some evidence that the facilities and other stakeholders i.e. the community or any local partner take ownership and responsibility to support the intervention	2 = There is some evidence that stakeholders have the ability to use their power to make decisions, advocate and support initiative	2 = There is evidence of some level of partnerships, collaborations and networks to support and sustain the intervention e.g. with some local stakeholders	2 = There is evidence of some level of acceptance, enjoyment and reward among stakeholders from participating in intervention e.g. Among direct beneficiaries staff and patients	2 = There is evidence of some level of involvement and participation of stakeholders who are affected by the intervention e.g. staff, patients	2 = There is evidence of some level of participation of community members to direct and shape the intervention to reflect their values, expectations and needs involving community groups e.g. involving lay workers from the community	2 = There is evidence of some level of involvement of patients in the intervention's processes, to understand patient's perspective e.g. adapting the process	2 = There is evidence of some level of involvement of staff in the planning, design, delivery and maintenance of the intervention
1 = There is little or no evidence of an influential patient, or group of people who have the ability and skills to advocate, communicate and support other patients at the facility level during the intervention.	1 = There is little or no evidence that any stakeholder take ownership and responsibility to support the intervention maybe just the facilities	1 = There is little or no evidence of the ability of stakeholders to use their power to make decisions, advocate and support initiative	1 = There is little or no evidence of partnerships, collaborations and networks to support and sustain the intervention e.g. with the facility or institution (s)	1 = There is little or no evidence of acceptance, enjoyment and reward among any stakeholder from participating in intervention	1 = There is little or no evidence of the involvement and participation of stakeholders who are affected by the intervention e.g. patients	1 = There is little or no evidence of participation of community membersto direct and shape the intervention to reflect their values, expectations and needs involving community groups e.g. community only involved as passive recipients	1 = There is little or no evidence of the involvement of patients in the intervention's processes e.g. only as passive participants	1 = There is evidence of involvement of staff in the delivery and maintenance of the intervention

Challenges reported	Comments (Pros & Cons)	Patients perspective	Staff perspective
Any challenges documented during implementation	Any gains or flaws highlighted by staff or patients during implementation	Any experiences or opinions expressed by patients	Any experiences or opinions expressed by staff

For peer review only

Reporting checklist for protocol of a systematic review.

Based on the PRISMA-P guidelines.

Instructions to authors

Complete this checklist by entering the page numbers from your manuscript where readers will find each of the items listed below.

Your article may not currently address all the items on the checklist. Please modify your text to include the missing information. If you are certain that an item does not apply, please write "n/a" and provide a short explanation.

Upload your completed checklist as an extra file when you submit to a journal.

In your methods section, say that you used the PRISMA-Preporting guidelines, and cite them as:

Moher D, Shamseer L, Clarke M, Ghersi D, Liberati A, Petticrew M, Shekelle P, Stewart LA. Preferred Reporting Items for Systematic Review and Meta-Analysis Protocols (PRISMA-P) 2015 statement. Syst Rev. 2015;4(1):1.

		Reporting Item	Page Number
**Title**			
Identification	#1a	Identify the report as a protocol of a systematic review	1
Update	#1b	If the protocol is for an update of a previous systematic review, identify as such	n/a

For peer review only

**Registration**			
	#2	If registered, provide the name of the registry (such as PROSPERO) and registration number	2
**Authors**			
Contact	#3a	Provide name, institutional affiliation, e-mail address of all protocol authors; provide physical mailing address of corresponding author	1
Contribution	#3b	Describe contributions of protocol authors and identify the guarantor of the review	12
**Amendments**			
	#4	If the protocol represents an amendment of a previously completed or published protocol, identify as such and list changes; otherwise, state plan for documenting important protocol amendments	n/a
**Support**			
Sources	#5a	Indicate sources of financial or other support for the review	12
Sponsor	#5b	Provide name for the review funder and / or sponsor	12
Role of sponsor or funder	#5c	Describe roles of funder(s), sponsor(s), and / or institution(s), if any, in developing the protocol	12
**Introduction**			

For peer review only

Rationale	#6	Describe the rationale for the review in the context of what is already known	5
Objectives	#7	Provide an explicit statement of the question(s) the review will address with reference to participants, interventions, comparators, and outcomes (PICO)	6
**Methods**			
Eligibility criteria	#8	Specify the study characteristics (such as PICO, study design, setting, time frame) and report characteristics (such as years considered, language, publication status) to be used as criteria for eligibility for the review	7
Information sources	#9	Describe all intended information sources (such as electronic databases, contact with study authors, trial registers or other grey literature sources) with planned dates of coverage	6
Search strategy	#10	Present draft of search strategy to be used for at least one electronic database, including planned limits, such that it could be repeated	6
Study records - data management	#11a	Describe the mechanism(s) that will be used to manage records and data throughout the review	6
Study records - selection process	#11b	State the process that will be used for selecting studies (such as two independent reviewers) through each phase of the review (that is, screening, eligibility and inclusion in meta-analysis)	7

For peer review only

Study records - data collection process	#11c	Describe planned method of extracting data from reports (such as piloting forms, done independently, in duplicate), any processes for obtaining and confirming data from investigators	7
Data items	#12	List and define all variables for which data will be sought (such as PICO items, funding sources), any pre-planned data assumptions and simplifications	7
Outcomes and prioritization	#13	List and define all outcomes for which data will be sought, including prioritization of main and additional outcomes, with rationale	8-10
Risk of bias in individual studies	#14	Describe anticipated methods for assessing risk of bias of individual studies, including whether this will be done at the outcome or study level, or both; state how this information will be used in data synthesis	11
Data synthesis	#15a	Describe criteria under which study data will be quantitatively synthesised	11
Data synthesis	#15b	If data are appropriate for quantitative synthesis, describe planned summary measures, methods of handling data and methods of combining data from studies, including any planned exploration of consistency (such as I2, Kendall's τ)	11
Data synthesis	#15c	Describe any proposed additional analyses (such as sensitivity or subgroup analyses, meta-regression)	11

For peer review only

Data synthesis	#15d	If quantitative synthesis is not appropriate, describe the type of summary planned	11
Meta-bias(es)	#16	Specify any planned assessment of meta-bias(es) (such as publication bias across studies, selective reporting within studies)	11
Confidence in cumulative evidence	#17	Describe how the strength of the body of evidence will be assessed (such as GRADE)	11

The PRISMA-P checklist is distributed under the terms of the Creative Commons Attribution License CC-BY 4.0. This checklist was completed on 21. July 2019 using https://www.goodreports.org/, a tool made by the EQUATOR Network in collaboration with Penelope.ai

For peer review only
